# Characterization of the Two-Speed Subgenomes of *Fusarium graminearum* Reveals the Fast-Speed Subgenome Specialized for Adaption and Infection

**DOI:** 10.3389/fpls.2017.00140

**Published:** 2017-02-14

**Authors:** Qinhu Wang, Cong Jiang, Chenfang Wang, Changjun Chen, Jin-Rong Xu, Huiquan Liu

**Affiliations:** ^1^State Key Laboratory of Crop Stress Biology for Arid Areas and College of Plant Protection, Northwest A&F UniversityYangling, China; ^2^College of Plant Protection, Nanjing Agricultural UniversityNanjing, China; ^3^Department of Botany and Plant Pathology, Purdue University, West LafayetteIN, USA

**Keywords:** Fusarium head blight, genomic variation, two-speed genome, positive selection, heterochromatin

## Abstract

Fusarium head blight, caused by *Fusarium graminearum*, is one of the most severe diseases on wheat and barley worldwide. Although the genomic data of several strains were published, the intragenomic variation of *F. graminearum* was not well characterized. Here, we sequenced three Chinese strains and conducted genome-wide comparisons. Our data revealed that all the sequenced strains were distinct from each other and over 350 genes were functionally lost in each of them. Variants of each strain were unevenly distributed in a highly conserved pattern along the chromosomes, resulting in a conserved two-speed genome. The fast subgenome has a lower GC content, shorter gene length, and higher variation of exon numbers than the slow subgenome. Genes related to interaction and pathogenicity, under positive selection, and up-regulated *in planta* were all significantly enriched in the fast subgenome. Furthermore, we found that the fast subgenome coincided with facultative heterochromatin regions that were repressed in vegetative stage but activated during infection as measured by RNA-seq and ChIP-seq data, suggesting that the fast subgenome is epigenetically regulated. Taken together, our data demonstrated that *F. graminearum* has a highly conserved two-speed genome and the fast subgenome responsible for adaption and infection is under the control of heterochromatin.

## Introduction

Wheat is one of the most cultivated staple crops that feed the world. Fusarium head blight (FHB), caused by *Fusarium graminearum*, is a great threat to the yields and quality of wheat, barley, and maize that directly relates to the survival and heath of human beings (Goswami and Kistler, 2004). *F. graminearum* can also generate mycotoxins, including deoxynivalenol and zearalenone that contaminates the infested kernels, which are harmful to humans and livestock (D’Mello et al., 1999; Hussein and Brasel, 2001; Haggblom and Nordkvist, 2015). In China, FHB was first reported in 1936, and its recent epidemics occurred in 2003, 2008, 2010, and 2012 (Zhang et al., 2012; Mehta, 2014). In particular, the FHB outbreak in 2012 is extremely destructive and over one-third of the wheat growing areas were affected (Mehta, 2014).

Decoding the genome is the first step for understanding the whole machinery for fungi development, infection, and spreading. The whole genome sequencing of *F. graminearum* American strain PH-1 revealed that the pathogen has very few repeats sequences and much more transcription factors and hydrolytic enzymes than other fungi (Cuomo et al., 2007). Intra-species comparison of an American strain GZ3639, which was shotgun sequenced with only 0.4-fold coverage, with the PH-1 revealed that the high single-nucleotide polymorphism (SNP) regions were correlated with pathogen specialization and mainly located in the telomeric or sub-telomeric regions (Cuomo et al., 2007). Comparative genomics of different species of *Fusarium* showed that the lineage-specific regions in the genome of *Fusarium oxysporum* were responsible for pathogenicity (Ma et al., 2010). Further studies also showed that the *Fusarium* pathogens have the bipartite (two-speed) genome architecture (Zhao et al., 2014; Sperschneider et al., 2015) as what happened in many other pathogens (Dong et al., 2015), such as oomycete pathogen *Phytophthora infestans* (Haas et al., 2009) and fungal pathogen *Leptosphaeria maculans* (Dong et al., 2015).

*Fusarium graminearum* is a worldwide pathogen, population analyses showed that even in a local region, the isolates of *F. graminearum* are very diverse (Qu et al., 2008; Yang et al., 2008; Zhang et al., 2012; Talas and McDonald, 2015; van der Lee et al., 2015). Besides the genome of strain PH-1 (Cuomo et al., 2007; King et al., 2015), recently, the resequencing data of three strains isolated from Australia (Gardiner et al., 2014), America and Canada (Walkowiak et al., 2015) were published. However, whether these strains have a different or conserved genomic variation remains largely unknown. Furthermore, no Chinese strains of *F. graminearum* were reported although great differentiations were existed (Qu et al., 2008; Yang et al., 2008; Zhang et al., 2012; van der Lee et al., 2015). We thus began our work by sequencing three Chinese strains isolated from main wheat growing regions to survey the genomic variations of *F. graminearum*. We performed comparative genomics with the previously reported strains to characterize the two-speed genome of *F. graminearum*. We also carried out RNA-seq analysis to determine the gene expression differences in the two subgenomes during plant infection. Moreover, we explored the differences of selection pressures and histone modification acting on the two subgenomes. Our study revealed that the two-speed genome of *F. graminearum* is highly conserved among different strains, and the fast-speed subgenome drove adaptive evolution and infection by heterochromatin regulation.

## Results

### Great Diversification of the Resequenced Genomes of *F. graminearum*

**FIGURE 1:**
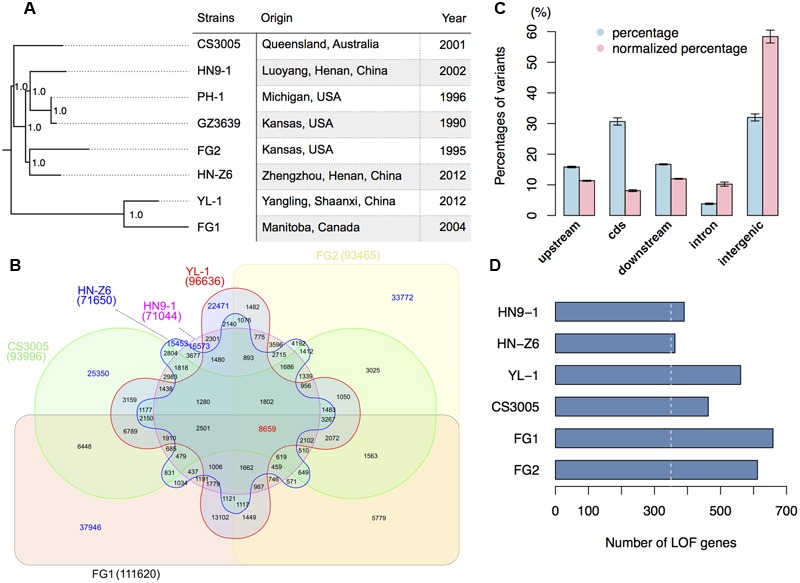
**Genomic variation of *F. graminearum* strains. (A)** Strains used in this study and their maximum likelihood phylogenetic tree based on SNP data. The tree was mid-point rooted. Numbers near the nodes indicate the branch support values (“1.0” means 100%) generated by SH-like approximate likelihood ratio test (SH-aLRT). **(B)** Venn diagram showing the common and specific SNPs among different strains in comparison with the reference genome sequence of PH-1. Digit in the brackets indicates the total number of SNPs for each strain. **(C)** Bar chart showing the proportions of variants located in different genomic regions. The percentages of variants in different genomic regions are in blue, while the percentages of variants in different genomic regions normalized by the total length of the corresponding regions are in red. The upstream and downstream sequences (represent UTRs) are the 500-bp 5′ and 3′ flanking sequences of the coding region (cds) of individual genes. Error bars represent the standard deviations of the proportions among the six strains. **(D)** Number of genes that have predicted (in blue) loss-of-function (LOF) variants in the six strains. The white-dashed line marks 350.

We resequenced three *F. graminearum* strains, HN9-1, HN-Z6, and YL-1 (**Figure 1A**) that were isolated from the main wheat growing regions in China. Along with the recently resequenced Australian strain CS3005 (Gardiner et al., 2014), Canadian strain FG1 (Walkowiak et al., 2015), and US strain FG2 (Walkowiak et al., 2015), we compared these six genomes to the reference genome of strain PH-1 isolated from Michigan, USA (Cuomo et al., 2007) (Supplementary Table S1). For each strain, about 95,157 ± 17,471 variants (**Table 1**) were called. In these variants, SNPs were the dominant genomic variations (94.3%). Small insertion and deletion variations (INDELs) only account for a small proportion of the total variants (**Table 1**). On average, the *F. graminearum* genome has 2.5 ± 0.4 SNPs per kb.

**Table 1:** Number of genomic variants identified in *F. graminearum* strains.

Strains	SNP	INDEL	Total variants
HN9-1	71,044	3,598	74,642
HN-Z6	71,650	3,582	75,232
YL-1	96,636	5,436	102,072
CS3005	93,996	5,136	99,132
FG1	111,620	8,512	120,132
FG2	93,465	6,265	99,730
GZ3639	10,304	–	–

With the 10,304 SNPs detected in the 0.4-fold genome sequence of *F. graminearum* strain GZ3639 (Cuomo et al., 2007; King et al., 2015), we have a total of 275,641 SNP sites compared to PH-1. SNP based phylogenomic analysis revealed that the strains from one country were not clustered together. For examples, the Chinese strain HN9-1 is clustered with the two US strains PH-1 and GZ3639 (**Figure 1A**). The two nationwide FHB outbreak strains YL-1 and HN-Z6 of China are most closely related to Canadian strain FG1 and US strain FG2, respectively (**Figure 1A**). While the Australian strain CS3005 is distant from all the other strains we analyzed here (**Figure 1A**). Furthermore, the number of unique SNPs in the resequenced strains, ranged from 15,453 to 37,946, is greater than any of the interaction numbers of the SNPs among FG1, FG2, CS3005, YL-1, HN9-1, and HN-Z6 (**Figure 1B**). These results consistent with the previous population studies that great genetic diversification exists in *F. graminearum* isolates (Qu et al., 2008; Yang et al., 2008; Zhang et al., 2012; Talas and McDonald, 2015; van der Lee et al., 2015).

### Over 350 Genes Were Functionally Lost in Each of the Resequenced Strains

When being mapped onto different genomic features, most variants of the resequenced strains are in the intergenic (32.2%) and coding regions (30.7%) (**Figure 1C**, blue column). Another one-third of them are in the 5′ or 3′ untranslated regions (UTRs; **Figure 1C**, blue column). To remove the potential effects of total lengths on the proportion of different genomic features, we normalized the variants proportions by the relative sizes of different genomic features. We found that the intergenic regions have a high degree of variation; the UTRs and intron have a comparable variation density, both higher than the coding regions but lower than the intergenic regions (**Figure 1C**, red column). This result indicates that the coding regions of *F. graminearum* were under strong purifying selection.

For the variants in the coding regions, 41.0% of them are missense variations. Remarkably, relative to the genome sequence of strain PH-1, a total of 4,994 variants (**Table 2**) caused start codon lost, stop codon gained, or frameshift, which potentially result in gene function losses. These variations affect 1,647 protein-coding genes, and for each strain, over 350 genes were functionally lost (**Figure 1D**). Among them, 41 genes were lost in all resequenced strains, while over 90 genes were specifically lost in each of the resequenced strains (Supplementary Figure S1). In addition, we identified 381 variants resulting in stop codon lost (**Table 2**) in the six resequenced strains that may affect translation and protein functions by adding a stretch of extra peptides to the C-terminal end.

**Table 2:** Number of variants that potentially result in gene loss-of-function.

Strains	Start lost	Stop gained	Frameshift	Stop lost
HN9-1	38	199	299	47
HN-Z6	29	180	287	46
YL-1	54	301	416	66
CS3005	40	272	360	65
FG1	56	346	842	84
FG2	40	333	902	73

### *Fusarium graminearum* Has a Highly Conserved Two-Speed Genome

**FIGURE 2:**
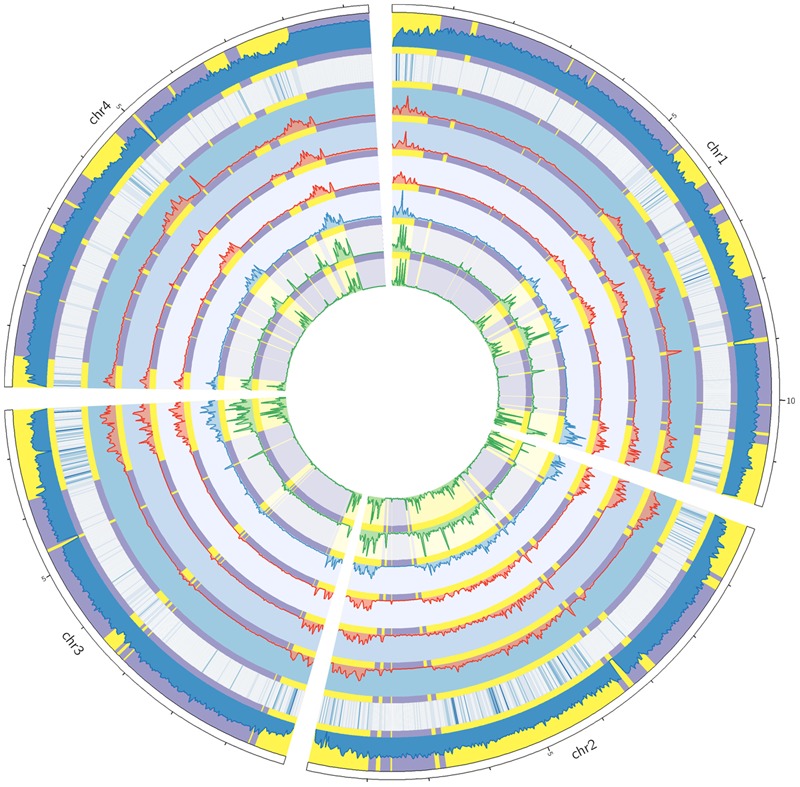
**Circos plot showing the variant distribution and conserved two-speed genome.** From the circus outside to inside are the four chromosomes of *F. graminearum*, histogram of GC contents, heat map of secreted protein genes, and variation densities of strains YL-1 (in red), HN9-1 (in red), HN-Z6 (in red), CS3005 (in blue), FG1 (in green), and FG2 (in green). The fast subgenome regions (highlighted in yellow) and the slow subgenome regions (highlighted in purple) are calculated by *depmixS4*.

To study the distributions of genomic variations, we calculated the variant frequencies in different chromosomal regions. For all sequenced strains, the variants were unevenly distributed along the four chromosomes (**Figure 2**). The variants were often enriched in specific chromosomal regions, especially in the telomeric or sub-telomeric regions (**Figure 2**). And this is consistent with the previous genome comparison between two American strains PH-1 and GZ3639 (Cuomo et al., 2007), and the inter-species comparison among *Fusarium* pathogens (Zhao et al., 2014; Sperschneider et al., 2015). Interestingly, the distribution patterns of variants were well conserved across all these strains (**Figure 2**), even in the specific SNP regions of each strain (Supplementary Figure S2).

**FIGURE 3:**
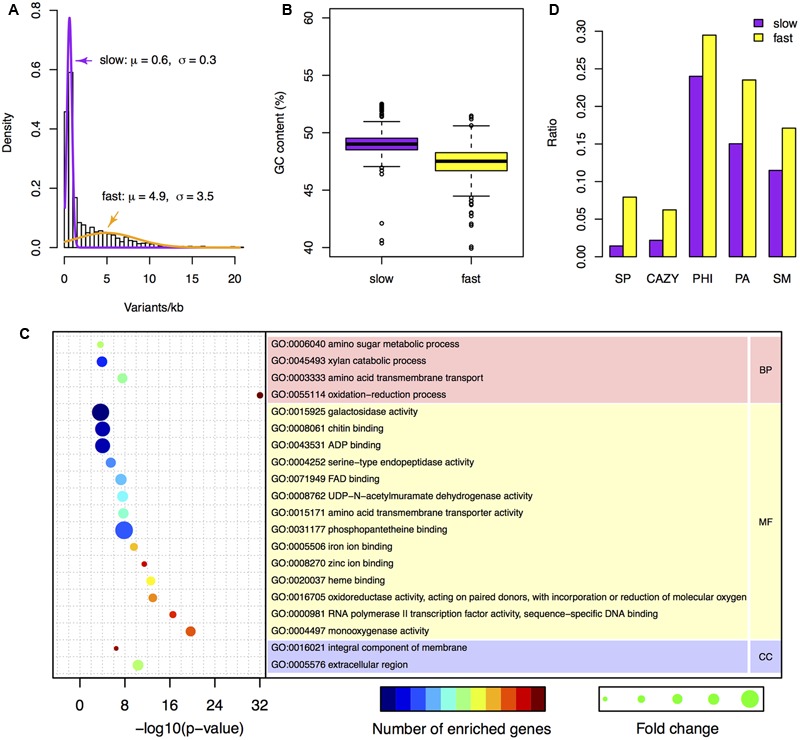
**Characteristics of the two-speed genome of *F. graminearum*. (A)** The probability density of the number of variants per kb among *F. graminearum* strains. The average numbers of variants per 25 kb in the six resequenced strains were used for modeling. Purple and orange curves represent the estimated probability distributions of the variants number for the slow and fast subgenome, respectively. **(B)** Boxplot showing the GC content of the fast and slow subgenomes. **(C)** GO enrichment analysis with the genes located in the fast subgenome of *F. graminearum*. BP, MF, and CC stand for biological process, molecular function, and cellular component, respectively. **(D)** The proportions of carbohydrate-active enzymes (CAZY) genes, pathogen–host interactions (PHI) genes, pathogen-associated (PA) genes, secondary metabolite (SM) genes, and secreted protein (SP) genes in the fast and slow subgenomes.

To analyze the variant distribution patterns in *F. graminearum*, we modeled the frequencies of variants (defined as number of variants per kb) with the expectation–maximization algorithm (Benaglia et al., 2009) by using all the variants identified in the six well-sequenced strains. The results clearly showed that the genome could be divided into two subgenomes: a fast subgenome with high frequency of variants and a slow subgenome with low frequency of variants (**Figure 3A**), similar to previous results of inter-species genome comparison (Zhao et al., 2014; Sperschneider et al., 2015). The rates for the fast and slow subgenomes were estimated at 4.9 ± 3.5 and 0.6 ± 0.3 variants per kb, respectively (**Figure 3A**). We used Viterbi algorithm (Visser and Speekenbrink, 2010) to determine the exact regions of the fast and slow speed subgenomes in *F. graminearum* (Supplementary Table S2). The results revealed that the fast subgenome contains 6,353 genes in 15.3 Mb region (**Figure 2**, highlighted in yellow), and the slow subgenome contains 7,811 genes in 22.7 Mb region (**Figure 2**, highlighted in purple).

### The Two Subgenomes of *F. graminearum* Have Distinct GC Content but Similar Gene Density

To investigate the differences between the two subgenomes, we calculated the GC content in each interval of the two subgenomes. Intriguingly, the fast subgenome has a GC content of 46.8%, which is significantly (*p*-value = 7.9e-31) lower than the slow subgenome (49.1%) (**Figures 2** and **3B**). Consistent with this observation, the GC content is lower for genes located in the fast subgenome (50.5%) than those in the slow subgenome (52.2%) (*p*-value = 4.0e-207, Supplementary Figure S3A). In addition, the gene length in fast subgenome region is slight shorter (*p*-value = 7.0e-3) than that in the slow subgenome region (Supplementary Figure S3B), and the exon number variation in the fast subgenome is much higher (*p*-value = 0) than that in the slow subgenome (Supplementary Figure S3C). We found that there are 415.2 and 344.1 genes per Mb in the fast and slow subgenomes, respectively. Thus the fast subgenome in *F. graminearum* does not coincide with the gene sparse region as what has been reported in repeat-rich oomycete pathogen *P. infestans* (Haas et al., 2009). Consistently, the gene borders, which consist of 5′ and 3′ flanking intergenic regions (FIRs), have a similar distribution in the fast and slow subgenomes of *F. graminearum*, which is also different with the two subgenomes of *P. infestans* (Haas et al., 2009; Supplementary Figure S4) and fungal pathogen *L. maculans* (Dong et al., 2015).

### The Fast Subgenome of *F. graminearum* Is Enriched for Genes Related to Interaction and Pathogenicity

To study the function of the fast subgenome, we performed gene ontology (GO) enrichment analysis. The fast subgenome is enriched for genes involving in xylan catabolic, amino acid transmembrane transport, amino sugar metabolic, and oxidation–reduction processes (**Figure 3C**). The proteins located in extracellular region were also overrepresented, suggesting that the fast subgenome may associate with the secreted proteins (SP). We thus compared the composition of SP genes in the two subgenomes. The completed *F. graminearum* genome has a total of 616 genes that encode SP (Cuomo et al., 2007). Of these, 504 (81.8%) are located in the fast subgenome, whereas only 112 (18.2%) are located in the slow subgenome. Thus, the fast subgenome is significantly (504/6353 ÷ 112/7811 = 5.5-fold, *p*-value = 5.5e-83) enriched for SP genes (**Figures 2** and **3D**). Consistent with this result, the carbohydrate-active enzymes (CAZY; Henrissat and Davies, 1997) that required for the degradation of plant cell wall to facilitate infection and/or gain nutrition were 2.8-fold enriched in the fast subgenome (**Figure 3D**). Furthermore, we found that the known pathogen–host interactions (PHI) genes (Urban et al., 2015), pathogen-associated (PA) genes (Sperschneider et al., 2013), and secondary metabolite (SM) genes (Sieber et al., 2014) are all overrepresented in the fast-speed subgenome (**Figure 3D**). These results suggest that the fast-speed subgenome of *F. graminearum* is enriched for interaction and pathogenicity related genes.

### The Fast Subgenome of *F. graminearum* Is Enriched for Genes under Positive Selection

**FIGURE 4:**
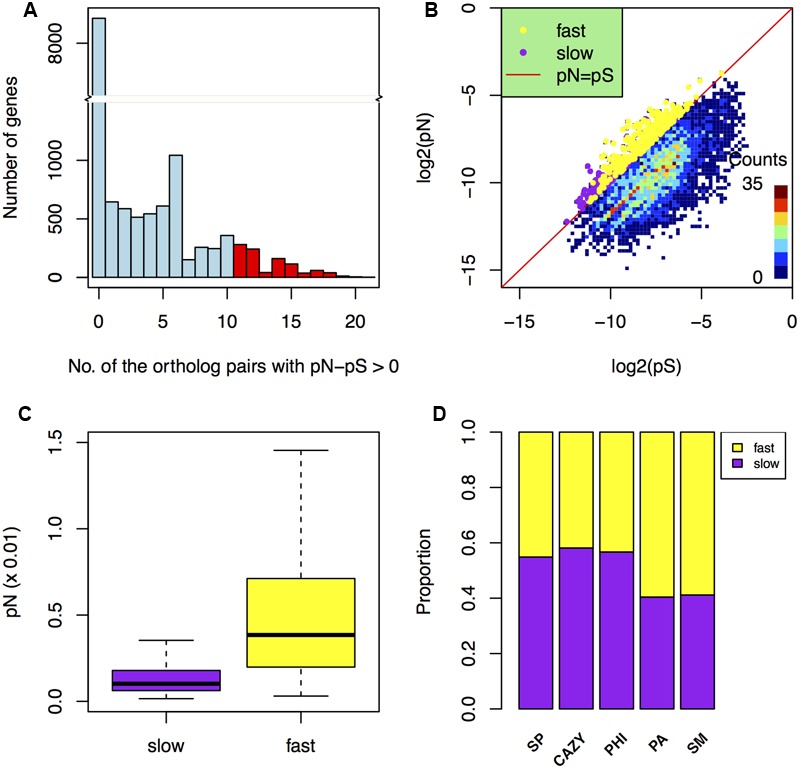
**Comparison of positive selection in the fast and slow subgenomes. (A)** Histogram showing the number of genes in the categories that grouped by the total number of ortholog pairs with pN–pS > 0. The red columns indicate the positive selected genes (screened by pN > pS in at least 11 ortholog pairs). **(B)** The distribution of the positively selected genes (screened by the average pN/pS > 1 and the average pS > 0) in the fast and slow subgenomes. The *x*-axis and *y*-axis are the logarithm of pS and pN, respectively. **(C)** Comparison of non-synonymous rates (Langmead et al., 2009) in the fast and slow subgenomes. **(D)** The proportions of carbohydrate-active enzymes (CAZY) genes, pathogen–host interactions (PHI) genes, pathogen-associated (PA) genes, secondary metabolite (SM) genes, and secreted protein (SP) genes that underwent positive selection relative to the total number of them located in the fast or slow subgenome.

To determine whether genes in the two subgenomes are underwent different selection pressures, we calculated the number of synonymous differences per synonymous site (pS) and the number of non-synonymous differences per non-synonymous site (pN) for individual *F. graminearum* genes with SNPs. By using two different methods (see Materials and Methods), a total of 1181 candidate of positive selected genes were found (**Figures 4A,B**). Among them, 609 genes (9.6%) are in the fast subgenome, which is 1.3-fold higher (*p*-value = 7.9e-7) than the rest 572 genes (7.3%) in the slow subgenome.

Additionally, the non-synonymous difference value pN in the fast subgenome is much higher (*p*-value = 4.8e-47) than that in the slow subgenome (**Figure 4C**). Furthermore, we found that the PA and SM genes in the fast subgenome contained more positively selected genes compared to that in the slow subgenome (**Figure 4D**), when normalized to the number of PA or SM genes in the each subgenomes. Positive selection can relate to different adaptation processes, such as environmental, geographical, host response. Therefore, our data further suggest that the fast subgenome of *F. graminearum* is enriched for genes required for adaptation.

### The Fast Subgenome of *F. graminearum* Is Enriched for Genes Up-Regulated *In planta*

**FIGURE 5:**
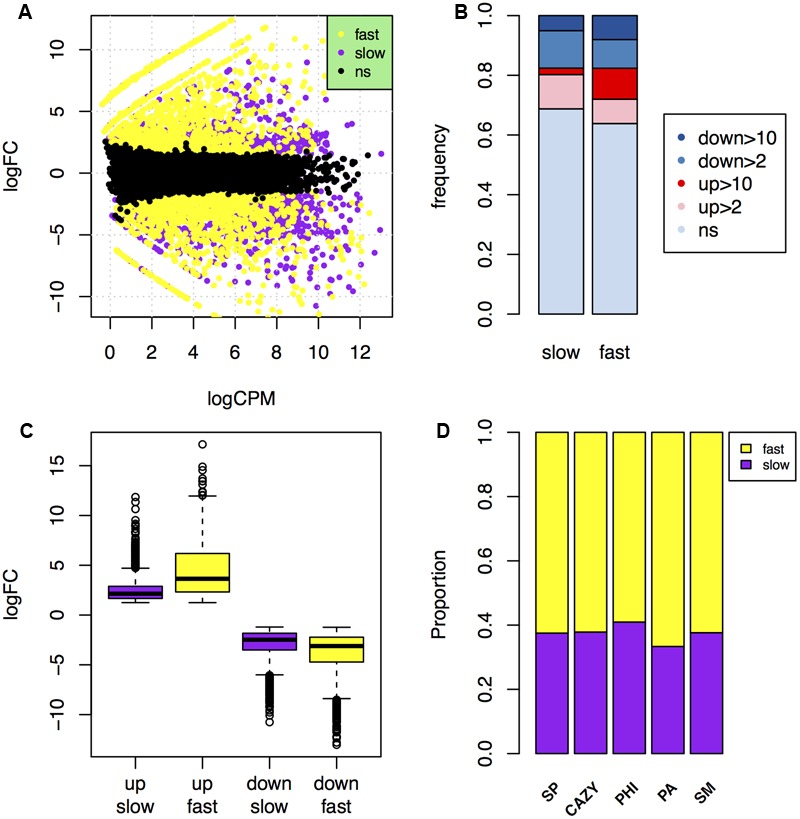
**Comparison of *in planta* gene expression in the fast and slow subgenomes. (A)** MA-plot of genes that were up- or down-regulated over 2-fold during plant infection in the fast (yellow) and slow (purple) subgenomes. **(B)** Comparison of the frequency of genes up- or down-regulated over 2- or 10-fold *in planta* in the fast and slow subgenomes. ns, not significant. **(C)** Comparison of the fold change levels of the up- and down-regulated genes in the fast and slow subgenomes. **(D)** The proportions of carbohydrate-active enzymes (CAZY) genes, pathogen–host interactions (PHI) genes, pathogen-associated (PA) genes, secondary metabolite (SM) genes, and secreted protein (SP) genes that *in planta* up-regulated relative to the total number of them located in the fast or slow subgenome.

To determine differences in the gene expressions in the fast and slow subgenomes during infection, we sequenced the samples of 6 days post-inoculated wheat heads, and performed RNA-seq analysis to find the differently expressed genes (DEGs) *in planta*. Of the 14,164 reference genes, 10,853 (76.6%) have at least one count per million in each of the two biological repeats. By using the gene expression data of mycelia as a background, a total of 4,737 genes (33.4%) were found to be differently expressed during plant infection, including 2,243 genes (15.8%) up-regulated and 2,494 genes (17.6%) down-regulated over two folds (**Table 3**; **Figure 5A**).

**Table 3:** The number of DEGs during plant infection for strain PH-1.

DEGs	Fast subgenome (6,353 genes)	Slow subgenome (7,811 genes)	*p*-value	Total
Up-regulated	1,177 (18.5%)	1,066 (13.6%)	1.8e-15	2,243
Down-regulated	1,120 (17.6%)	1,374 (17.6%)	0.48	2,494
Total	2,297 (36.2%)	2,440 (31.2%)	4.0e-10	4,737

About 36.2% genes (2,297 genes) are differently expressed in the fast subgenome, which is significantly higher than the 31.2% DEGs (2,440 genes) in the slow subgenome (**Table 3**). Interestingly, this difference is absolutely caused by the up-regulation of genes in the fast subgenome, since the genes down-regulated have no significant differences (*p*-value = 0.48) between the two subgenomes (**Table 3**; **Figure 5B**). There are 1177 (18.5%) and 1066 (13.6%) up-regulated genes in the fast and slow subgenome, respectively (**Table 3**; **Figure 5B**). Moreover, the number of extremely up-regulated genes (≥10-fold) in the fast subgenome is 4.8-fold enrichment than that in the slow subgenome (**Figure 5B**). Notably, the extent of gene up-regulation and down-regulation is much higher (1.8-fold with *p*-value = 8.5e-92 for up-regulated DEGs, 1.3-fold with *p*-value = 7.3e-28 for down-regulated DEGs) in the fast subgenome than in the slow subgenome (**Figure 5C**), indicating that the fast subgenome may contains more genes that are involved in rapid response to host plants. Notably, the SP, CAZY, PHI, PA, and SM genes in the fast subgenome contained more genes up-regulated *in planta* compared to that in the slow subgenome (**Figure 5D**). All these data suggest that the fast subgenome of *F. graminearum* play important roles for plant infection.

### The Fast Subgenome of *F. graminearum* Is Associated with Facultative Heterochromatin

**FIGURE 6:**
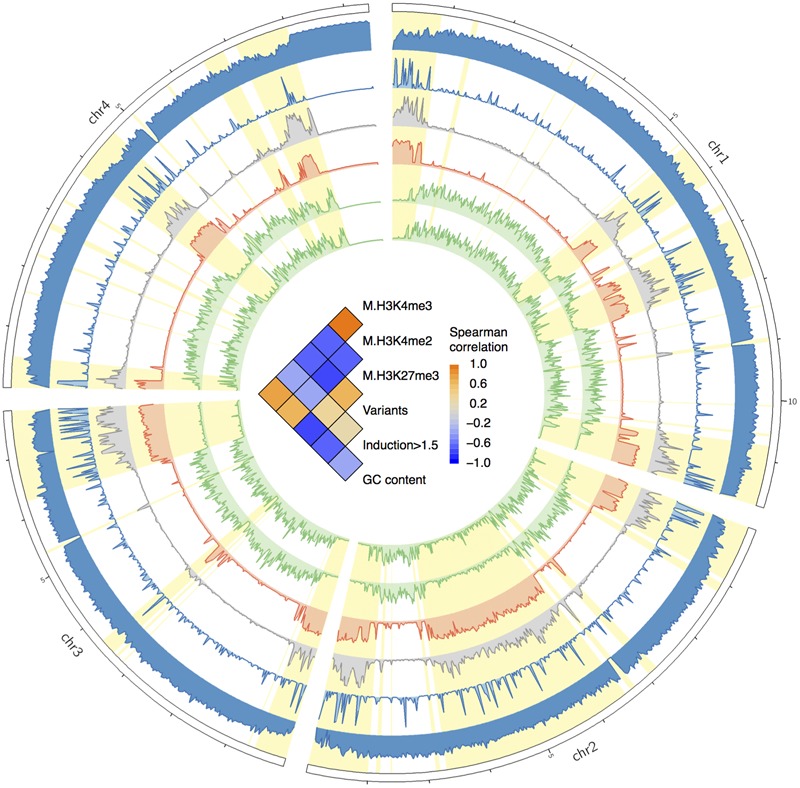
**Circos plot showing the distributions of GC contents, variants, genes upregulated *in planta*, and histone marks.** From the circus outside to inside are the histogram of GC contents, gene expression fold changes *in planta*, variants frequency, and frequencies of histone mark of H3K27me3, H3K4me2, and H3K4me3. The fast subgenome region is highlighted in yellow. In the center of the circus, the heat-matrix shows the Spearman correlation coefficients between different tracks.

It have been recognized that the regulation of massive concerted expression of pathogen genes during infection is chromatin-based, and these genes are located in the low GC isochores (Soyer et al., 2015). Hence, the rapid up-regulation of the genes in the fast subgenome is likely epigenetically programmed by chromatin. To verify if the fast subgenome is associated with the plastic heterochromatin in *F. graminearum*, we mapped the facultative heterochromatin mark H3K27me3 associated ChIP-seq reads generated by Connolly et al. (2013) to the genome. Interestingly, the fast subgenome is overwhelmingly located in the facultative heterochromatin regions with high level of H3K27me3 marks (**Figure 6**, red track, highlighted in yellow). In contrast, the activation marks (H3K4me2, H3K4me3) are co-localized with the slow subgenome (**Figure 6**, green tracks). Therefore, the fast subgenome is correlated with the facultative heterochromatin represented by the repressive H3K27me3 mark, and the slow subgenome is correlated with the euchromatin represented by the activating H3K4me2/H3K4me3 mark. All these observed correlations were further verified by Spearman correlation analysis (**Figure 6**, inner correlation heatmap).

### Identification of Candidate Genes Responsible for *Fusarium*–Wheat Interaction

**FIGURE 7:**
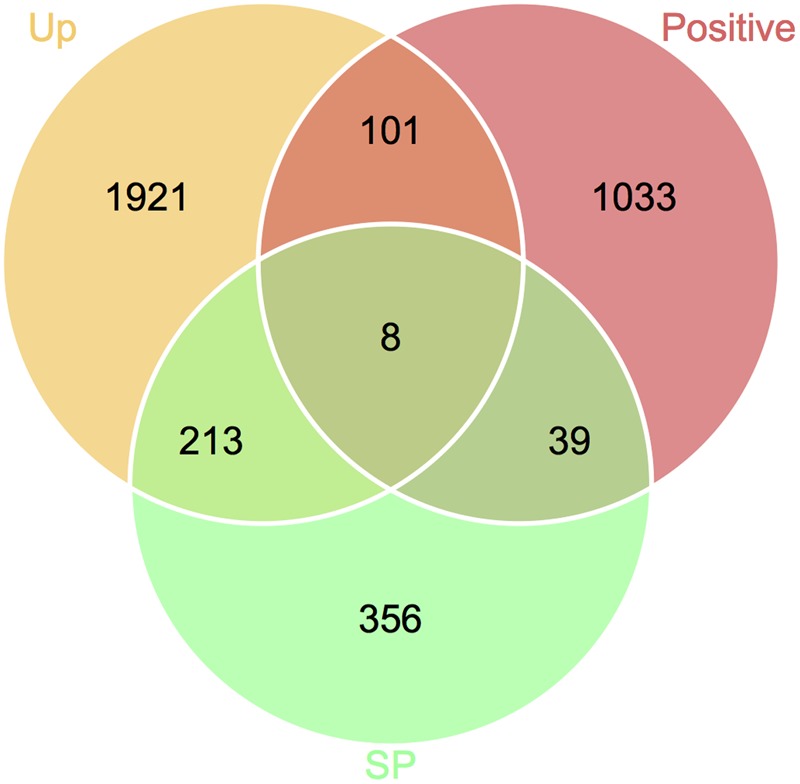
**Venn diagram showing the candidate genes responsible for *Fusarium*–wheat interaction.** Genes that are up-regulated during plant infection (Up), under positive selection (Positive), and predicted to encode secreted proteins (SP) were used for screening.

To define genes responsible for *Fusarium*–wheat interaction, we considered the three points following. First, to battle with plant, a gene involved in infection directly is likely to be up-regulated. Second, if a gene is really important for pathogenicity, during the co-evolution of host and pathogen, it should bear with significant positive selection. Third, some of the genes (such as effector genes) involved in infection may secrete to the extracellular space or enter plant cell to facilitate infection. By applying these criteria, eight genes responsible for *Fusarium*–wheat interaction were identified (**Figure 7**; **Table 4**). Interestingly, six of these eight genes are in the fast subgenome while only two belong to the slow subgenome. In these genes, five of them have PHI database hits, including three genes can result reduced pathogen virulence upon interruption (**Table 4**), and one PA gene (Sperschneider et al., 2013) defined previously (**Table 4**). Interestingly, two of the candidates (FGRRES_10712 and FGRRES_15917_M) have a high sequence similarity with the effector candidates identified by interspecies comparison previously (Sperschneider et al., 2015), including a homolog of FGRRES_10999, which have underwent diversifying selection among species, but no SNPs were found within the eight strains analyzed in this study. Therefore, our intraspecies comparison has not only extended the effector candidates, but also supplied good candidates for recently active effectors.

**Table 4:** Candidate genes responsible for *Fusarium*–wheat interaction.

Gene ID	Length (aa)	Subgenome	PHI phenotype	Annotation
FGRRES_00006^∗^	296	Fast	Reduced virulence	Gegh 16 protein
FGRRES_00061	125	Fast		Killer kp4
FGRRES_00184	314	Fast		Xylanase
FGRRES_06733	716	Slow	Unaffected pathogenicity	Catalase
FGRRES_10712^§^	395	Fast	Reduced virulence	Alkaline Protease
FGRRES_10713	557	Fast	Unaffected pathogenicity	Lipase 2
FGRRES_13515^∗#^	122	Fast		Unknown
FGRRES_15917_M^†^	276	Slow	Reduced virulence	Xylanase

*^∗^Have loss-of-function variation found in resequenced strains. ^#^Identified as one of the pathogen-associated gene that described in Sperschneider et al. (2013). ^§^Homologous to the effector candidate FGSG_03315 identified by interspecies genome comparison (Sperschneider et al., 2015). ^†^Homologous to the effector candidate FGSG_10999 identified by interspecies genome comparison (Sperschneider et al., 2015).*

## Discussion

Previous studies have shown that *F. graminearum* has a two-speed genome (Zhao et al., 2014; Sperschneider et al., 2015) and the highly variable regions are responsible for pathogen specialization (Cuomo et al., 2007). However, it is not clear whether the highly variable regions are conserved among different *F. graminearum* strains. In this study, we resequenced three Chinese strains of *F. graminearum* and performed comparative genomics of them with three American strains (Cuomo et al., 2007; Walkowiak et al., 2015), one Canadian strain (Walkowiak et al., 2015), and one Australian strain (Gardiner et al., 2014). Although great divergences exist in different strains, our data showed that the two-speed genome of *F. graminearum* is highly conserved among different strains. The fast subgenome has a lower GC content than the slow subgenome. All the interaction and pathogenicity related genes, including SP, CAZY, PHI, PA, and SM genes, were overrepresented in the fast subgenome. In addition, genes underwent positive selection and/or up-regulated *in planta* were also enriched in the fast subgenomes of *F. graminearum*. These results suggest that the fast subgenome drives adaptive evolution and infection in *F. graminearum*.

We showed that the features of the two-speed genome of *F. graminearum* are different from that of fungal pathogen *L. maculans* (Grandaubert et al., 2014) and oomycete pathogen *P. infestans* (Haas et al., 2009). First, the two subgenomes of *F. graminearum* have a comparable subgenome sizes and number of genes, while the two subgenomes of *L. maculans* (Grandaubert et al., 2014) and *P. infestans* (Haas et al., 2009) are very different. Second, the fast subgenomes of *L. maculans* and *P. infestans* (Haas et al., 2009; Grandaubert et al., 2014; Dong et al., 2015) were coincided with the gene sparse regions. In contrast, the gene density in the fast subgenome of *F. graminearum* is slightly higher than the slow subgenome in *F. graminearum*. Furthermore, the fast subgenome has a slightly shorter gene length with larger exon number variation than the slow subgenome. Thus, the fast subgenome of *F. graminearum* may be a little bit more compact than the slow subgenome. These differences maybe due to very little of repetitive sequences in *F. graminearum* genome (Cuomo et al., 2007; King et al., 2015).

The distinct two-speed genome model of *F. graminearum* described here indicates that, although the diverse eukaryotic pathogens may have different origins for the fast subgenome, they are convergently evolved a subgenome for adaptive selection. The classical two-speed genome of plant pathogens is driven by repetitive sequences (Raffaele et al., 2010; Grandaubert et al., 2014; Dong et al., 2015). For now, it is not clear about the potential mechanism of two-speed genome evolved in *F. graminearum*. However, one common feature of the fast subgenomes in *F. graminearum, L. maculans*, and *P. infestans* is their AT-rich characteristic. Chromatin with high AT isochores (Soyer et al., 2015) is proposed as a concise and parsimony approach for the concerted expression of infection-related genes. In addition, the non-conserved region (relevant to the fast subgenome) of *F. graminearum* genome has weak gene expression in mycelia but has a highly variable gene expression between mycelia and conidia stages (Zhao et al., 2014). In this study, we further demonstrated that the fast subgenome has higher variable gene expression *in planta* and *in vitro*. Therefore, the true difference under the two subgenomes may be lying on the chromatin structures.

We analyzed the ChIP-seq data generated before (Connolly et al., 2013), and the result showed a perfect correlation between H3K27me3 and the fast subgenome. Thus the fast subgenome is the heterochromatin in hyphae stage, and H3K27me3 is likely to be the silencing mark. Once infection to be or being established, the fast subgenome is reprogrammed to facility the rapid responses of infection and adaptation. In fact, heterochromatin is shown to regulate secondary metabolism genes and effectors in fungal pathogens *F. graminearum, Fusarium verticillioides*, and *L. maculans* (Reyes-Dominguez et al., 2012; Visentin et al., 2012; Soyer et al., 2014, 2015). Our study also showed that the fast subgenome is enriched for interaction and infection related genes, positive selected genes, and secondary metabolism genes, thus heterochromatin-based regulation of the fast subgenome is required for the infection and adaptation in *F. graminearum*.

## Materials and Methods

### Fungal Strains and Growth Condition

*Fusarium graminearum* strains HN9-1 (kindly provided by Dr. Zhonghua Ma, Zhejiang University), HN-Z6 (kindly provided by Dr. Wenming Zheng, Henan Agricultural University), and YL-1 (this study) isolated from wheat head with FHB in China were maintained on the potato dextrose agar medium at 25°C. The strains (available upon request) were deposited at the Northwest A&F University - Purdue University Joint Research Center.

### Genome Resequencing Analysis

For the Chinese strains HN9-1, HN-Z6, and YL-1, paired ends 90-bp high throughput DNA sequencing (Supplementary Table S1) was performed at Beijing Genomics Institution (Shenzhen, China). The resequencing data were deposited at NCBI SRA database under accession number SRP063887. The raw reads of Australian strain CS3005 (Gardiner et al., 2014) were downloaded from NCBI SRA database. The reads were mapped on the reference genome of strain PH-1 (King et al., 2015) by Bowtie 2 (Langmead and Salzberg, 2012) and variants were called by SAMtools (Li et al., 2009) with its default parameters. For Canadian strain FG1 and American strain FG2 (Walkowiak et al., 2015), the whole genome shotgun assembly (no raw data available) were used. To call precise variants, the contigs were reordered by ABACAS (Assefa et al., 2009) with the reference genome of strain PH-1 (King et al., 2015), and MUMmer (Delcher et al., 1999) were used to calling the variants with its default parameters.

For the phylogenomic analysis, all the SNP data were converted to segregating sites, and PhyML (Guindon et al., 2010) was used to build the maximum likelihood tree with its default parameters. The Venn diagram of SNPs and functionally lost genes was draw by InteractiVenn (Heberle et al., 2015). The annotation of variation was performed by snpEff (Cingolani et al., 2012). To visualize the genomic variation data, Circos (Krzywinski et al., 2009) were used. For the density calculation of variants, SP and GC contents, the entire genome was split to 25 kb intervals. To model the genomic variations and estimate the variation rates, *mixtools* (Benaglia et al., 2009) developed in R were used. The hidden Markov states of each interval were determined by using R package *depmixS4* (Visser and Speekenbrink, 2010). The gene length, exon numbers, and FIRs analysis were extracted or calculated from the completed genome annotation (King et al., 2015) by custom Perl scripts. GO enrichment analysis was performed with Blast2GO (Conesa et al., 2005), and the *p*-values were adjusted with Benjamini–Hochberg procedure by controlling false discovery rate (FDR) to 0.05. All the Perl, R, and Shell scripts used in this study for resequencing and other analysis were available on GitHub^1^.

### Positive Selection Analysis

To evaluate the selection pressures across different strains of *F. graminearum*, each SNP in the genes were used to generate an alternative sequence in all the resequenced strains analyzed here. Since only SNP data were used, the alignment of the sequences is thus codon-aligned. The proportions of synonymous and non-synonymous (Langmead et al., 2009) differences per sites (Nei and Gojobori, 1986) were calculated by SNAP (Ganeshan et al., 1997). Two different methods were used to screen the positive selected genes (pN > pS) from the 21 pairwise comparisons of the pN and pS values in the seven strains (PH-1, YL-1, HN9-1, HN-Z6, CS3005, FG1, and FG2). First, if a gene has a larger pN value than pS value in more than 50% of the total 21 pairwise comparisons (i.e., at least 11 pairwise), it is regarded as a positive selected gene. Second, if the average pN value of a gene is larger than its average pS value that is not 0, it is also regarded as a positive selected gene.

### RNA-Seq Analysis

For sampling, freshly harvested PH-1 conidia from carboxymethyl cellulose cultures were re-suspended to a final concentration of 10^5^ spores per milliliter in sterile distilled water. Flowering wheat heads of cultivar XiaoYan 22 were drop-inoculated with 10 μl of conidium suspensions as described previously (Jiang et al., 2015). Total RNAs were extracted with TRIzol (Invitrogen, USA) and treated with RNase-free DNase I. RNA-seq libraries with the average insert size of 300 bp were constructed as described in the manufacture. Illumina deep sequencing with paired-end 2 × 125 bp model were performed at Novogene Bioinformatics Technology (Beijing, China). RNA-seq data were deposited at NCBI SRA database under accession number SRP063766. The mycelia RNA-seq data were downloaded from NCBI SRA database under accession number SRP060552, which were generated by our lab previously using the same protocol. The paired end clean reads (Supplementary Table S1) were mapped to the *F. graminearum* reference genome (Cuomo et al., 2007; King et al., 2015) by hisat (Kim et al., 2015) and the abundances of the gene expression were count by using featureCounts (Liao et al., 2014). The differences of gene expression between the infection tissue and mycelia with two biological repeats were calculated with R package *edgeRun* (Dimont et al., 2015). A gene with log_2_FC (log_2_ fold change) greater than 1 and FDR less than 0.05 was regarded as DEGs.

### ChIP-Seq Analysis

The *F. graminearum* ChIP-seq (chromatin immunoprecipitation and high throughput DNA sequencing) data (Connolly et al., 2013) of H3K4me2 (SRR999613, SRR999614, SRR999615, SRR999616), H3K4me3 (SRR999617, SRR999618), and H3K27me3 (SRR999608, SRR999609, SRR999610) from mycelia were downloaded from NCBI SRA database. They were mapped on strain PH-1 reference genome via Bowtie (Langmead et al., 2009). The mapped bam files from the high and low nitrogen conditions were merged with SAMtools (Li et al., 2009) and the coverage was calculated with BEDtools (Quinlan and Hall, 2010). For correlation analysis, Spearman correlation developed in R was used. The ChIP-seq and relevant RNA-seq data were visualized by Circos (Krzywinski et al., 2009).

### Pathogenicity and Adaption Related Genes

For CAZY genes (Henrissat and Davies, 1997) in *F. graminearum*, Zhao et al. (2013) dataset without glycosyltransferases (irrelevant to pathogenicity) were used for analysis. *F. graminearum* genes have a significant hit (E-value cut-off = 1e-5) against PHI database (Urban et al., 2015) were defined as PHI genes. For PA genes, Sperschneider et al. (2013) datasets were used. The SM genes in *F. graminearum* were described by Sieber et al. (2014). For SP, the candidates developed in the completed genome of *F. graminearum* by Brown et al. (2012) algorithm were used. These SP have excluded the ones containing transmembrane domains or GPI anchors, and the subcellular localization was further evaluated by WolfPsort.

### Statistical Tests

One-sided *t*-tests were applied to compare the difference of GC content, gene length, pN, and gene up-regulation/down-regulation in the fast and slow subgenomes. One-sided Fisher’s exact tests were used to compare the numbers of SP, positive selection genes, up-regulated/down-regulated genes, and infection-related genes in the two-speed subgenomes. To access the variations difference of the exon number in the two-speed subgenomes, *F*-test was performed. All the statistical tests and related graphics were performed and illustrated with R.

## Author Contributions

HL and J-RX conceived and designed the experiments; QW, HL, CJ, CC, and CW performed the analysis; QW, HL, and J-RX wrote the manuscript.

## Conflict of Interest Statement

The authors declare that the research was conducted in the absence of any commercial or financial relationships that could be construed as a potential conflict of interest.

## Footnotes

1 https://github.com/wangqinhu/fgreseq
