# Supplementary material for: Characterization of the Two-Speed Subgenomes of Fusarium graminearum Reveals the Fast-Speed Subgenome Specialized for Adaption and Infection
Source: Front Plant Sci. 2017 Feb 14;8:140. doi: 10.3389/fpls.2017.00140 (PMC5306128; doi:10.3389/fpls.2017.00140)
Supplement: Supplementary file 1 [file Data_Sheet_1.pdf]

# *Supplementary Material*

# Characterization of the Two-speed Subgenomes of *Fusarium graminearum* Reveals the Fast-speed Subgenome Specialized for Adaption and Infection

**Qinhu Wang, Cong Jiang, Chenfang Wang, Changjun Chen, Jin-Rong Xu, and Huiquan Liu***

*** Correspondence:** Huiquan Liu: liuhuiquan@nwsuaf.edu.cn

## 1 Supplementary Figures

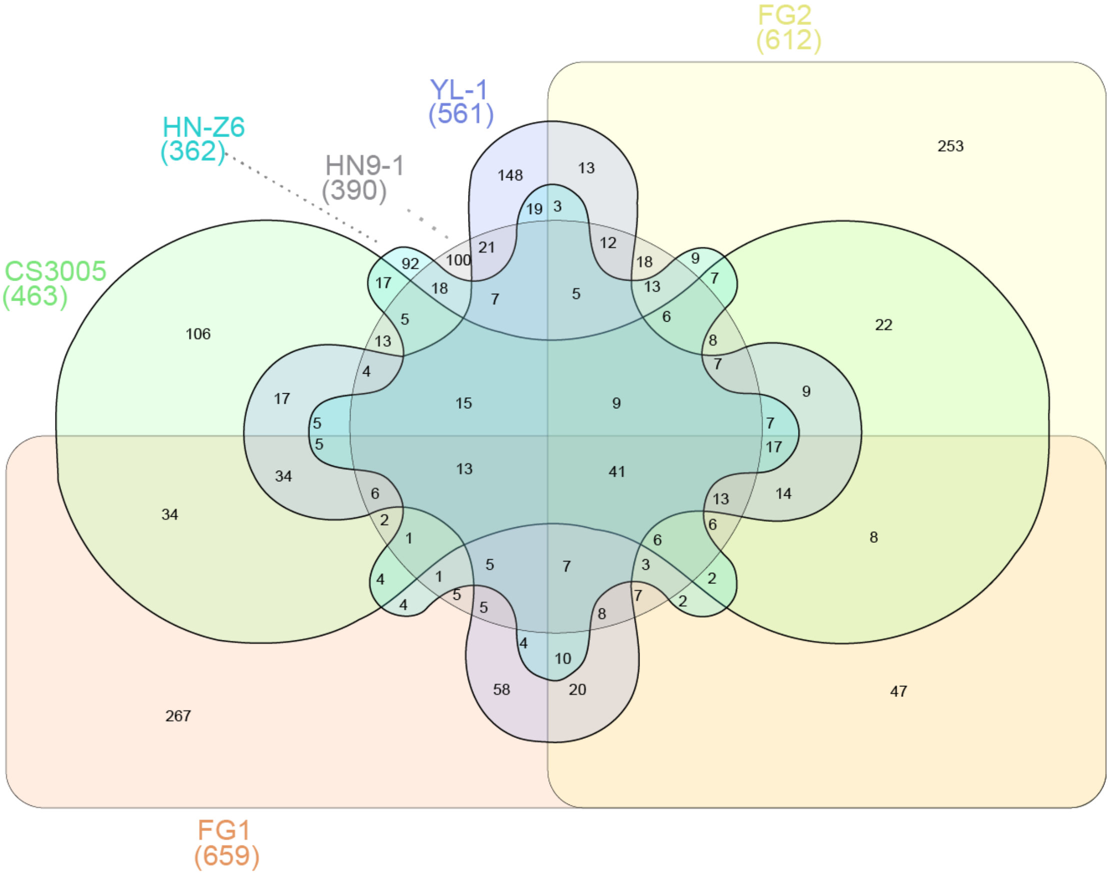


**Supplementary Figure S1.** Venn diagram showing the common and specific loss-of-function genes among the six resequenced strains.

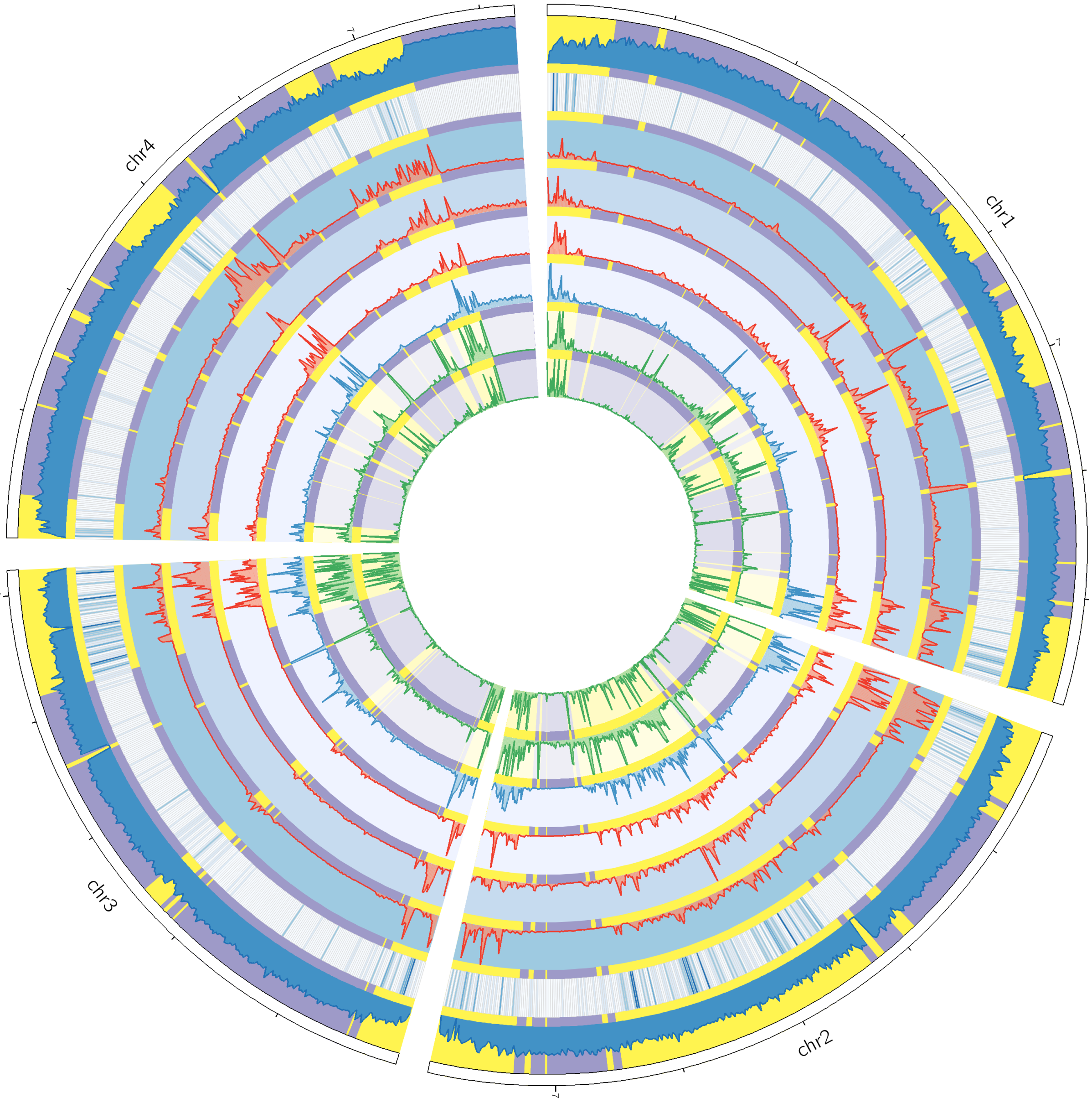


**Supplementary Figure S2.** Circos plot showing the strain-specific SNPs distributions in the two subgenomes. From the circus outside to inside are the four chromosomes of *F. graminearum*, histogram of GC contents, heat map of secreted protein genes, and strain-specific SNPs densities of YL-1 (in red), HN9-1 (in red), HN-Z6 (in red), CS3005 (in blue), FG1 (in green), and FG2 (in green). The fast subgenome regions (highlighted in yellow) and the slow subgenome regions (highlighted in purple) are calculated by *depmixS4*.

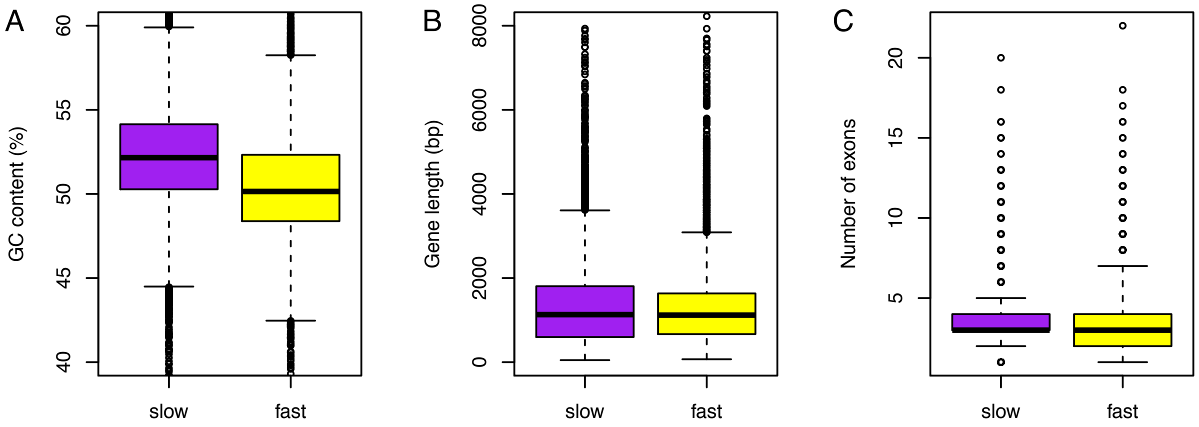


**Supplementary Figure S3.** Boxplot showing the differences of GC content (A), length (B), and exon number (C) of genes in the two subgenomes of *F. graminearum*.

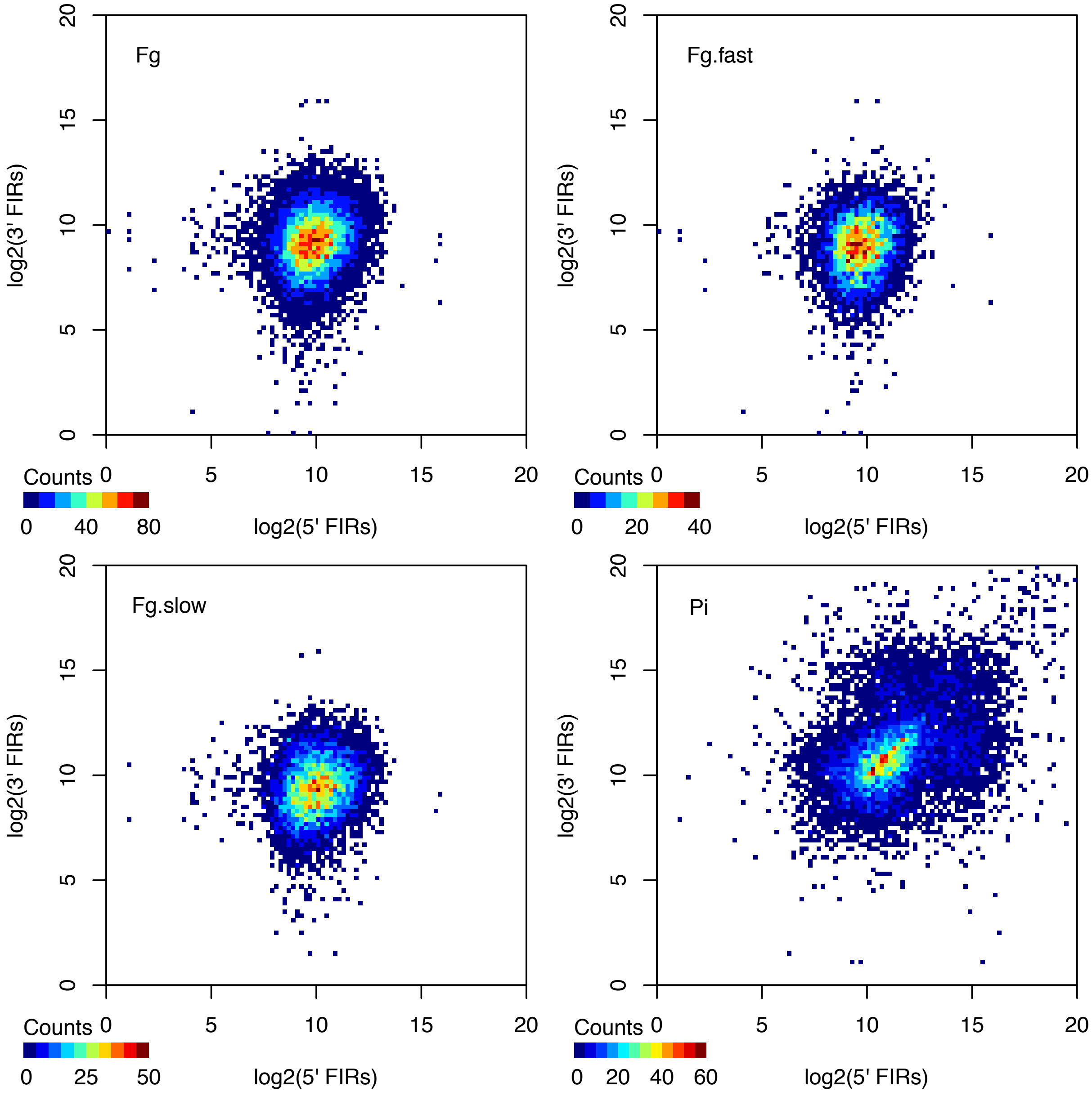


**Supplementary Figure S4. Distributions of gene border lengths in the two subgenomes of *F. graminearum*.** The lengths of each pair of 5' and 3' flanking intergenic regions (FIR) represent the borders of a gene were used to measure the gene density. The x-axis and y-axis are the logarithm of 5' FIR and 3' FIR, respectively. Fg stands for *F. graminearum*, Fg.fast and Fg.slow stand for the fast and slow sub-genome of *F. graminearum*, respectively. Pi stands for *P. infestans* that serves as the positive control for a species known to have gene-sparse and gene-dense genomic regions.

## 2 Supplementary Tables

**Supplementary Table S1.** Statistics of the deep sequencing data used in this study.

| | Type | Total reads | Length (bp) | Coverage | Accession number |
|---|---|---|---|---|---|
| HN9-1 § | WGS | 11,944,445 | 90 x 2 | 57.3 | PRJNA296400 |
| HN-Z6 § | WGS | 11,111,112 | 90 x 2 | 53.4 | PRJNA296400 |
| YL-1 § | WGS | 11,111,112 | 90 x 2 | 52.8 | PRJNA296400 |
| CS3005 | WGS | 10,592,131 | 100 x 2 | 54.1 | SRR2308812 |
| FG1 | WGS | - | - | 144.0 | LAKA01000000 |
| FG2 | WGS | - | - | 86.0 | LAJZ01000000 |
| Mycelia.1 | RNA-seq | 9,107,356 | 125 x 2 | - | SRR2485276 |
| Mycelia.2 | RNA-seq | 9,158,410 | 125 x 2 | - | SRR2518132 |
| Infection.1 § | RNA-seq | 45,150,628 | 125 x 2 | - | PRJNA295929 |
| Infection.2 § | RNA-seq | 45,809,397 | 125 x 2 | - | PRJNA295929 |
| H3K4me2.1 | ChIP-seq | 15,545,387 | 53 | - | SRR999613 |
| H3K4me2.2 | ChIP-seq | 12,851,288 | 53 | - | SRR999614 |
| H3K4me2.3 | ChIP-seq | 1,513,114 | 51 | - | SRR999615 |
| H3K4me2.4 | ChIP-seq | 8,569,375 | 51 | - | SRR999616 |
| H3K4me3.1 | ChIP-seq | 22,563,631 | 51 | - | SRR999617 |
| H3K4me3.2 | ChIP-seq | 24,505,737 | 51 | - | SRR999618 |
| H3K27me3.1 | ChIP-seq | 14,983,721 | 53 | - | SRR999608 |
| H3K27me3.2 | ChIP-seq | 6,871,011 | 53 | - | SRR999609 |
| H3K27me3.3 | ChIP-seq | 5,110,400 | 51 | - | SRR999610 |

§ Sequenced by this study.

**Supplementary Table S2.** The detail regions of the fast and slow sub genomes.

| Chr | Start | End | Subgenome |
|---|---|---|---|
| 1 | 1 | 25000 | fast |
| 1 | 25001 | 50000 | fast |
| 1 | 50001 | 75000 | fast |
| 1 | 75001 | 100000 | fast |
| 1 | 100001 | 125000 | fast |
| 1 | 125001 | 150000 | fast |
| 1 | 150001 | 175000 | fast |
| 1 | 175001 | 200000 | fast |

| | | | |
|---|---|---|---|
| 1 | 200001 | 225000 | fast |
| 1 | 225001 | 250000 | fast |
| 1 | 250001 | 275000 | fast |
| 1 | 275001 | 300000 | fast |
| 1 | 300001 | 325000 | fast |
| 1 | 325001 | 350000 | fast |
| 1 | 350001 | 375000 | fast |
| 1 | 375001 | 400000 | fast |
| 1 | 400001 | 425000 | fast |
| 1 | 425001 | 450000 | fast |
| 1 | 450001 | 475000 | fast |
| 1 | 475001 | 500000 | fast |
| 1 | 500001 | 525000 | fast |
| 1 | 525001 | 550000 | fast |
| 1 | 550001 | 575000 | fast |
| 1 | 575001 | 600000 | fast |
| 1 | 600001 | 625000 | fast |
| 1 | 625001 | 650000 | fast |
| 1 | 650001 | 675000 | fast |
| 1 | 675001 | 700000 | fast |
| 1 | 700001 | 725000 | fast |
| 1 | 725001 | 750000 | fast |
| 1 | 750001 | 775000 | fast |
| 1 | 775001 | 800000 | fast |
| 1 | 800001 | 825000 | fast |
| 1 | 825001 | 850000 | slow |
| 1 | 850001 | 875000 | slow |
| 1 | 875001 | 900000 | slow |
| 1 | 900001 | 925000 | slow |
| 1 | 925001 | 950000 | slow |
| 1 | 950001 | 975000 | slow |
| 1 | 975001 | 1000000 | slow |
| 1 | 1000001 | 1025000 | slow |
| 1 | 1025001 | 1050000 | slow |
| 1 | 1050001 | 1075000 | slow |
| 1 | 1075001 | 1100000 | slow |
| 1 | 1100001 | 1125000 | slow |
| 1 | 1125001 | 1150000 | slow |
| 1 | 1150001 | 1175000 | slow |
| 1 | 1175001 | 1200000 | slow |
| 1 | 1200001 | 1225000 | slow |
| 1 | 1225001 | 1250000 | slow |
| 1 | 1250001 | 1275000 | slow |
| 1 | 1275001 | 1300000 | slow |
| 1 | 1300001 | 1325000 | slow |
| 1 | 1325001 | 1350000 | slow |
| 1 | 1350001 | 1375000 | fast |

| | | | |
|---|---|---|---|
| 1 | 1375001 | 1400000 | fast |
| 1 | 1400001 | 1425000 | fast |
| 1 | 1425001 | 1450000 | fast |
| 1 | 1450001 | 1475000 | slow |
| 1 | 1475001 | 1500000 | slow |
| 1 | 1500001 | 1525000 | slow |
| 1 | 1525001 | 1550000 | slow |
| 1 | 1550001 | 1575000 | slow |
| 1 | 1575001 | 1600000 | slow |
| 1 | 1600001 | 1625000 | slow |
| 1 | 1625001 | 1650000 | slow |
| 1 | 1650001 | 1675000 | slow |
| 1 | 1675001 | 1700000 | slow |
| 1 | 1700001 | 1725000 | slow |
| 1 | 1725001 | 1750000 | slow |
| 1 | 1750001 | 1775000 | slow |
| 1 | 1775001 | 1800000 | slow |
| 1 | 1800001 | 1825000 | slow |
| 1 | 1825001 | 1850000 | slow |
| 1 | 1850001 | 1875000 | slow |
| 1 | 1875001 | 1900000 | slow |
| 1 | 1900001 | 1925000 | slow |
| 1 | 1925001 | 1950000 | slow |
| 1 | 1950001 | 1975000 | slow |
| 1 | 1975001 | 2000000 | slow |
| 1 | 2000001 | 2025000 | slow |
| 1 | 2025001 | 2050000 | slow |
| 1 | 2050001 | 2075000 | slow |
| 1 | 2075001 | 2100000 | slow |
| 1 | 2100001 | 2125000 | slow |
| 1 | 2125001 | 2150000 | slow |
| 1 | 2150001 | 2175000 | slow |
| 1 | 2175001 | 2200000 | slow |
| 1 | 2200001 | 2225000 | slow |
| 1 | 2225001 | 2250000 | slow |
| 1 | 2250001 | 2275000 | slow |
| 1 | 2275001 | 2300000 | slow |
| 1 | 2300001 | 2325000 | slow |
| 1 | 2325001 | 2350000 | slow |
| 1 | 2350001 | 2375000 | slow |
| 1 | 2375001 | 2400000 | slow |
| 1 | 2400001 | 2425000 | slow |
| 1 | 2425001 | 2450000 | slow |
| 1 | 2450001 | 2475000 | slow |
| 1 | 2475001 | 2500000 | slow |
| 1 | 2500001 | 2525000 | slow |
| 1 | 2525001 | 2550000 | slow |
| 1 | 2550001 | 2575000 | slow |

| | | | |
|---|---|---|---|
| 1 | 2575001 | 2600000 | slow |
| 1 | 2600001 | 2625000 | slow |
| 1 | 2625001 | 2650000 | slow |
| 1 | 2650001 | 2675000 | slow |
| 1 | 2675001 | 2700000 | slow |
| 1 | 2700001 | 2725000 | slow |
| 1 | 2725001 | 2750000 | slow |
| 1 | 2750001 | 2775000 | slow |
| 1 | 2775001 | 2800000 | slow |
| 1 | 2800001 | 2825000 | slow |
| 1 | 2825001 | 2850000 | slow |
| 1 | 2850001 | 2875000 | slow |
| 1 | 2875001 | 2900000 | slow |
| 1 | 2900001 | 2925000 | slow |
| 1 | 2925001 | 2950000 | slow |
| 1 | 2950001 | 2975000 | slow |
| 1 | 2975001 | 3000000 | slow |
| 1 | 3000001 | 3025000 | slow |
| 1 | 3025001 | 3050000 | slow |
| 1 | 3050001 | 3075000 | slow |
| 1 | 3075001 | 3100000 | slow |
| 1 | 3100001 | 3125000 | slow |
| 1 | 3125001 | 3150000 | fast |
| 1 | 3150001 | 3175000 | slow |
| 1 | 3175001 | 3200000 | slow |
| 1 | 3200001 | 3225000 | slow |
| 1 | 3225001 | 3250000 | slow |
| 1 | 3250001 | 3275000 | slow |
| 1 | 3275001 | 3300000 | slow |
| 1 | 3300001 | 3325000 | slow |
| 1 | 3325001 | 3350000 | slow |
| 1 | 3350001 | 3375000 | slow |
| 1 | 3375001 | 3400000 | slow |
| 1 | 3400001 | 3425000 | slow |
| 1 | 3425001 | 3450000 | slow |
| 1 | 3450001 | 3475000 | slow |
| 1 | 3475001 | 3500000 | slow |
| 1 | 3500001 | 3525000 | slow |
| 1 | 3525001 | 3550000 | slow |
| 1 | 3550001 | 3575000 | fast |
| 1 | 3575001 | 3600000 | slow |
| 1 | 3600001 | 3625000 | slow |
| 1 | 3625001 | 3650000 | slow |
| 1 | 3650001 | 3675000 | slow |
| 1 | 3675001 | 3700000 | slow |
| 1 | 3700001 | 3725000 | slow |
| 1 | 3725001 | 3750000 | slow |

| | | | |
|---|---|---|---|
| 1 | 3750001 | 3775000 | slow |
| 1 | 3775001 | 3800000 | slow |
| 1 | 3800001 | 3825000 | slow |
| 1 | 3825001 | 3850000 | slow |
| 1 | 3850001 | 3875000 | slow |
| 1 | 3875001 | 3900000 | slow |
| 1 | 3900001 | 3925000 | slow |
| 1 | 3925001 | 3950000 | slow |
| 1 | 3950001 | 3975000 | slow |
| 1 | 3975001 | 4000000 | slow |
| 1 | 4000001 | 4025000 | slow |
| 1 | 4025001 | 4050000 | slow |
| 1 | 4050001 | 4075000 | slow |
| 1 | 4075001 | 4100000 | slow |
| 1 | 4100001 | 4125000 | slow |
| 1 | 4125001 | 4150000 | slow |
| 1 | 4150001 | 4175000 | slow |
| 1 | 4175001 | 4200000 | slow |
| 1 | 4200001 | 4225000 | slow |
| 1 | 4225001 | 4250000 | slow |
| 1 | 4250001 | 4275000 | slow |
| 1 | 4275001 | 4300000 | slow |
| 1 | 4300001 | 4325000 | slow |
| 1 | 4325001 | 4350000 | slow |
| 1 | 4350001 | 4375000 | slow |
| 1 | 4375001 | 4400000 | slow |
| 1 | 4400001 | 4425000 | slow |
| 1 | 4425001 | 4450000 | slow |
| 1 | 4450001 | 4475000 | slow |
| 1 | 4475001 | 4500000 | slow |
| 1 | 4500001 | 4525000 | slow |
| 1 | 4525001 | 4550000 | slow |
| 1 | 4550001 | 4575000 | slow |
| 1 | 4575001 | 4600000 | slow |
| 1 | 4600001 | 4625000 | slow |
| 1 | 4625001 | 4650000 | slow |
| 1 | 4650001 | 4675000 | slow |
| 1 | 4675001 | 4700000 | slow |
| 1 | 4700001 | 4725000 | slow |
| 1 | 4725001 | 4750000 | slow |
| 1 | 4750001 | 4775000 | slow |
| 1 | 4775001 | 4800000 | slow |
| 1 | 4800001 | 4825000 | slow |
| 1 | 4825001 | 4850000 | slow |
| 1 | 4850001 | 4875000 | slow |
| 1 | 4875001 | 4900000 | slow |
| 1 | 4900001 | 4925000 | slow |
| 1 | 4925001 | 4950000 | slow |

| | | | |
|---|---|---|---|
| 1 | 4950001 | 4975000 | slow |
| 1 | 4975001 | 5000000 | slow |
| 1 | 5000001 | 5025000 | slow |
| 1 | 5025001 | 5050000 | slow |
| 1 | 5050001 | 5075000 | slow |
| 1 | 5075001 | 5100000 | slow |
| 1 | 5100001 | 5125000 | slow |
| 1 | 5125001 | 5150000 | slow |
| 1 | 5150001 | 5175000 | slow |
| 1 | 5175001 | 5200000 | slow |
| 1 | 5200001 | 5225000 | slow |
| 1 | 5225001 | 5250000 | slow |
| 1 | 5250001 | 5275000 | slow |
| 1 | 5275001 | 5300000 | slow |
| 1 | 5300001 | 5325000 | slow |
| 1 | 5325001 | 5350000 | slow |
| 1 | 5350001 | 5375000 | slow |
| 1 | 5375001 | 5400000 | fast |
| 1 | 5400001 | 5425000 | slow |
| 1 | 5425001 | 5450000 | slow |
| 1 | 5450001 | 5475000 | slow |
| 1 | 5475001 | 5500000 | fast |
| 1 | 5500001 | 5525000 | fast |
| 1 | 5525001 | 5550000 | fast |
| 1 | 5550001 | 5575000 | fast |
| 1 | 5575001 | 5600000 | fast |
| 1 | 5600001 | 5625000 | fast |
| 1 | 5625001 | 5650000 | fast |
| 1 | 5650001 | 5675000 | fast |
| 1 | 5675001 | 5700000 | fast |
| 1 | 5700001 | 5725000 | fast |
| 1 | 5725001 | 5750000 | fast |
| 1 | 5750001 | 5775000 | fast |
| 1 | 5775001 | 5800000 | fast |
| 1 | 5800001 | 5825000 | fast |
| 1 | 5825001 | 5850000 | fast |
| 1 | 5850001 | 5875000 | fast |
| 1 | 5875001 | 5900000 | fast |
| 1 | 5900001 | 5925000 | fast |
| 1 | 5925001 | 5950000 | fast |
| 1 | 5950001 | 5975000 | fast |
| 1 | 5975001 | 6000000 | fast |
| 1 | 6000001 | 6025000 | fast |
| 1 | 6025001 | 6050000 | fast |
| 1 | 6050001 | 6075000 | fast |
| 1 | 6075001 | 6100000 | fast |
| 1 | 6100001 | 6125000 | fast |

| | | | |
|---|---|---|---|
| 1 | 6125001 | 6150000 | fast |
| 1 | 6150001 | 6175000 | fast |
| 1 | 6175001 | 6200000 | slow |
| 1 | 6200001 | 6225000 | slow |
| 1 | 6225001 | 6250000 | slow |
| 1 | 6250001 | 6275000 | slow |
| 1 | 6275001 | 6300000 | slow |
| 1 | 6300001 | 6325000 | slow |
| 1 | 6325001 | 6350000 | slow |
| 1 | 6350001 | 6375000 | slow |
| 1 | 6375001 | 6400000 | slow |
| 1 | 6400001 | 6425000 | slow |
| 1 | 6425001 | 6450000 | slow |
| 1 | 6450001 | 6475000 | slow |
| 1 | 6475001 | 6500000 | slow |
| 1 | 6500001 | 6525000 | slow |
| 1 | 6525001 | 6550000 | slow |
| 1 | 6550001 | 6575000 | slow |
| 1 | 6575001 | 6600000 | slow |
| 1 | 6600001 | 6625000 | fast |
| 1 | 6625001 | 6650000 | fast |
| 1 | 6650001 | 6675000 | fast |
| 1 | 6675001 | 6700000 | fast |
| 1 | 6700001 | 6725000 | slow |
| 1 | 6725001 | 6750000 | slow |
| 1 | 6750001 | 6775000 | slow |
| 1 | 6775001 | 6800000 | slow |
| 1 | 6800001 | 6825000 | slow |
| 1 | 6825001 | 6850000 | slow |
| 1 | 6850001 | 6875000 | slow |
| 1 | 6875001 | 6900000 | slow |
| 1 | 6900001 | 6925000 | fast |
| 1 | 6925001 | 6950000 | fast |
| 1 | 6950001 | 6975000 | fast |
| 1 | 6975001 | 7000000 | fast |
| 1 | 7000001 | 7025000 | fast |
| 1 | 7025001 | 7050000 | fast |
| 1 | 7050001 | 7075000 | fast |
| 1 | 7075001 | 7100000 | fast |
| 1 | 7100001 | 7125000 | fast |
| 1 | 7125001 | 7150000 | fast |
| 1 | 7150001 | 7175000 | fast |
| 1 | 7175001 | 7200000 | fast |
| 1 | 7200001 | 7225000 | fast |
| 1 | 7225001 | 7250000 | fast |
| 1 | 7250001 | 7275000 | fast |
| 1 | 7275001 | 7300000 | fast |
| 1 | 7300001 | 7325000 | fast |

| | | | |
|---|---|---|---|
| 1 | 7325001 | 7350000 | fast |
| 1 | 7350001 | 7375000 | fast |
| 1 | 7375001 | 7400000 | fast |
| 1 | 7400001 | 7425000 | fast |
| 1 | 7425001 | 7450000 | fast |
| 1 | 7450001 | 7475000 | fast |
| 1 | 7475001 | 7500000 | fast |
| 1 | 7500001 | 7525000 | fast |
| 1 | 7525001 | 7550000 | fast |
| 1 | 7550001 | 7575000 | fast |
| 1 | 7575001 | 7600000 | fast |
| 1 | 7600001 | 7625000 | fast |
| 1 | 7625001 | 7650000 | fast |
| 1 | 7650001 | 7675000 | fast |
| 1 | 7675001 | 7700000 | fast |
| 1 | 7700001 | 7725000 | fast |
| 1 | 7725001 | 7750000 | fast |
| 1 | 7750001 | 7775000 | fast |
| 1 | 7775001 | 7800000 | fast |
| 1 | 7800001 | 7825000 | fast |
| 1 | 7825001 | 7850000 | fast |
| 1 | 7850001 | 7875000 | fast |
| 1 | 7875001 | 7900000 | fast |
| 1 | 7900001 | 7925000 | slow |
| 1 | 7925001 | 7950000 | slow |
| 1 | 7950001 | 7975000 | slow |
| 1 | 7975001 | 8000000 | slow |
| 1 | 8000001 | 8025000 | slow |
| 1 | 8025001 | 8050000 | slow |
| 1 | 8050001 | 8075000 | slow |
| 1 | 8075001 | 8100000 | slow |
| 1 | 8100001 | 8125000 | slow |
| 1 | 8125001 | 8150000 | slow |
| 1 | 8150001 | 8175000 | slow |
| 1 | 8175001 | 8200000 | slow |
| 1 | 8200001 | 8225000 | slow |
| 1 | 8225001 | 8250000 | slow |
| 1 | 8250001 | 8275000 | slow |
| 1 | 8275001 | 8300000 | slow |
| 1 | 8300001 | 8325000 | slow |
| 1 | 8325001 | 8350000 | slow |
| 1 | 8350001 | 8375000 | slow |
| 1 | 8375001 | 8400000 | slow |
| 1 | 8400001 | 8425000 | fast |
| 1 | 8425001 | 8450000 | slow |
| 1 | 8450001 | 8475000 | slow |
| 1 | 8475001 | 8500000 | slow |

| | | | |
|---|---|---|---|
| 1 | 8500001 | 8525000 | slow |
| 1 | 8525001 | 8550000 | slow |
| 1 | 8550001 | 8575000 | slow |
| 1 | 8575001 | 8600000 | slow |
| 1 | 8600001 | 8625000 | slow |
| 1 | 8625001 | 8650000 | slow |
| 1 | 8650001 | 8675000 | slow |
| 1 | 8675001 | 8700000 | slow |
| 1 | 8700001 | 8725000 | slow |
| 1 | 8725001 | 8750000 | slow |
| 1 | 8750001 | 8775000 | slow |
| 1 | 8775001 | 8800000 | slow |
| 1 | 8800001 | 8825000 | slow |
| 1 | 8825001 | 8850000 | slow |
| 1 | 8850001 | 8875000 | slow |
| 1 | 8875001 | 8900000 | slow |
| 1 | 8900001 | 8925000 | slow |
| 1 | 8925001 | 8950000 | slow |
| 1 | 8950001 | 8975000 | fast |
| 1 | 8975001 | 9000000 | fast |
| 1 | 9000001 | 9025000 | fast |
| 1 | 9025001 | 9050000 | slow |
| 1 | 9050001 | 9075000 | slow |
| 1 | 9075001 | 9100000 | slow |
| 1 | 9100001 | 9125000 | slow |
| 1 | 9125001 | 9150000 | slow |
| 1 | 9150001 | 9175000 | slow |
| 1 | 9175001 | 9200000 | slow |
| 1 | 9200001 | 9225000 | slow |
| 1 | 9225001 | 9250000 | slow |
| 1 | 9250001 | 9275000 | slow |
| 1 | 9275001 | 9300000 | slow |
| 1 | 9300001 | 9325000 | slow |
| 1 | 9325001 | 9350000 | slow |
| 1 | 9350001 | 9375000 | slow |
| 1 | 9375001 | 9400000 | slow |
| 1 | 9400001 | 9425000 | slow |
| 1 | 9425001 | 9450000 | slow |
| 1 | 9450001 | 9475000 | slow |
| 1 | 9475001 | 9500000 | slow |
| 1 | 9500001 | 9525000 | slow |
| 1 | 9525001 | 9550000 | slow |
| 1 | 9550001 | 9575000 | slow |
| 1 | 9575001 | 9600000 | slow |
| 1 | 9600001 | 9625000 | slow |
| 1 | 9625001 | 9650000 | slow |
| 1 | 9650001 | 9675000 | slow |
| 1 | 9675001 | 9700000 | slow |

| | | | |
|---|---|---|---|
| 1 | 9700001 | 9725000 | slow |
| 1 | 9725001 | 9750000 | slow |
| 1 | 9750001 | 9775000 | slow |
| 1 | 9775001 | 9800000 | slow |
| 1 | 9800001 | 9825000 | slow |
| 1 | 9825001 | 9850000 | slow |
| 1 | 9850001 | 9875000 | slow |
| 1 | 9875001 | 9900000 | slow |
| 1 | 9900001 | 9925000 | slow |
| 1 | 9925001 | 9950000 | slow |
| 1 | 9950001 | 9975000 | slow |
| 1 | 9975001 | 10000000 | slow |
| 1 | 10000001 | 10025000 | slow |
| 1 | 10025001 | 10050000 | slow |
| 1 | 10050001 | 10075000 | slow |
| 1 | 10075001 | 10100000 | fast |
| 1 | 10100001 | 10125000 | slow |
| 1 | 10125001 | 10150000 | slow |
| 1 | 10150001 | 10175000 | slow |
| 1 | 10175001 | 10200000 | slow |
| 1 | 10200001 | 10225000 | slow |
| 1 | 10225001 | 10250000 | slow |
| 1 | 10250001 | 10275000 | slow |
| 1 | 10275001 | 10300000 | slow |
| 1 | 10300001 | 10325000 | slow |
| 1 | 10325001 | 10350000 | slow |
| 1 | 10350001 | 10375000 | slow |
| 1 | 10375001 | 10400000 | slow |
| 1 | 10400001 | 10425000 | slow |
| 1 | 10425001 | 10450000 | slow |
| 1 | 10450001 | 10475000 | slow |
| 1 | 10475001 | 10500000 | slow |
| 1 | 10500001 | 10525000 | slow |
| 1 | 10525001 | 10550000 | slow |
| 1 | 10550001 | 10575000 | fast |
| 1 | 10575001 | 10600000 | fast |
| 1 | 10600001 | 10625000 | slow |
| 1 | 10625001 | 10650000 | slow |
| 1 | 10650001 | 10675000 | slow |
| 1 | 10675001 | 10700000 | slow |
| 1 | 10700001 | 10725000 | slow |
| 1 | 10725001 | 10750000 | slow |
| 1 | 10750001 | 10775000 | fast |
| 1 | 10775001 | 10800000 | fast |
| 1 | 10800001 | 10825000 | fast |
| 1 | 10825001 | 10850000 | fast |
| 1 | 10850001 | 10875000 | fast |

| | | | |
|---|---|---|---|
| 1 | 10875001 | 10900000 | fast |
| 1 | 10900001 | 10925000 | fast |
| 1 | 10925001 | 10950000 | fast |
| 1 | 10950001 | 10975000 | fast |
| 1 | 10975001 | 11000000 | fast |
| 1 | 11000001 | 11025000 | fast |
| 1 | 11025001 | 11050000 | fast |
| 1 | 11050001 | 11075000 | fast |
| 1 | 11075001 | 11100000 | fast |
| 1 | 11100001 | 11125000 | fast |
| 1 | 11125001 | 11150000 | fast |
| 1 | 11150001 | 11175000 | fast |
| 1 | 11175001 | 11200000 | fast |
| 1 | 11200001 | 11225000 | fast |
| 1 | 11225001 | 11250000 | fast |
| 1 | 11250001 | 11275000 | fast |
| 1 | 11275001 | 11300000 | fast |
| 1 | 11300001 | 11325000 | fast |
| 1 | 11325001 | 11350000 | fast |
| 1 | 11350001 | 11375000 | fast |
| 1 | 11375001 | 11400000 | fast |
| 1 | 11400001 | 11425000 | fast |
| 1 | 11425001 | 11450000 | fast |
| 1 | 11450001 | 11475000 | fast |
| 1 | 11475001 | 11500000 | fast |
| 1 | 11500001 | 11525000 | fast |
| 1 | 11525001 | 11550000 | fast |
| 1 | 11550001 | 11575000 | fast |
| 1 | 11575001 | 11600000 | fast |
| 1 | 11600001 | 11625000 | fast |
| 1 | 11625001 | 11650000 | fast |
| 1 | 11650001 | 11675000 | fast |
| 1 | 11675001 | 11700000 | fast |
| 1 | 11700001 | 11725000 | fast |
| 1 | 11725001 | 11750000 | fast |
| 1 | 11750001 | 11775000 | fast |
| 2 | 1 | 25000 | fast |
| 2 | 25001 | 50000 | fast |
| 2 | 50001 | 75000 | fast |
| 2 | 75001 | 100000 | fast |
| 2 | 100001 | 125000 | fast |
| 2 | 125001 | 150000 | fast |
| 2 | 150001 | 175000 | fast |
| 2 | 175001 | 200000 | fast |
| 2 | 200001 | 225000 | fast |
| 2 | 225001 | 250000 | fast |
| 2 | 250001 | 275000 | fast |
| 2 | 275001 | 300000 | fast |

| | | | |
|---|---|---|---|
| 2 | 300001 | 325000 | fast |
| 2 | 325001 | 350000 | fast |
| 2 | 350001 | 375000 | fast |
| 2 | 375001 | 400000 | fast |
| 2 | 400001 | 425000 | fast |
| 2 | 425001 | 450000 | fast |
| 2 | 450001 | 475000 | fast |
| 2 | 475001 | 500000 | fast |
| 2 | 500001 | 525000 | fast |
| 2 | 525001 | 550000 | fast |
| 2 | 550001 | 575000 | fast |
| 2 | 575001 | 600000 | fast |
| 2 | 600001 | 625000 | fast |
| 2 | 625001 | 650000 | fast |
| 2 | 650001 | 675000 | fast |
| 2 | 675001 | 700000 | fast |
| 2 | 700001 | 725000 | fast |
| 2 | 725001 | 750000 | fast |
| 2 | 750001 | 775000 | fast |
| 2 | 775001 | 800000 | fast |
| 2 | 800001 | 825000 | fast |
| 2 | 825001 | 850000 | fast |
| 2 | 850001 | 875000 | fast |
| 2 | 875001 | 900000 | fast |
| 2 | 900001 | 925000 | fast |
| 2 | 925001 | 950000 | fast |
| 2 | 950001 | 975000 | slow |
| 2 | 975001 | 1000000 | slow |
| 2 | 1000001 | 1025000 | slow |
| 2 | 1025001 | 1050000 | fast |
| 2 | 1050001 | 1075000 | fast |
| 2 | 1075001 | 1100000 | fast |
| 2 | 1100001 | 1125000 | fast |
| 2 | 1125001 | 1150000 | fast |
| 2 | 1150001 | 1175000 | fast |
| 2 | 1175001 | 1200000 | slow |
| 2 | 1200001 | 1225000 | slow |
| 2 | 1225001 | 1250000 | slow |
| 2 | 1250001 | 1275000 | slow |
| 2 | 1275001 | 1300000 | slow |
| 2 | 1300001 | 1325000 | slow |
| 2 | 1325001 | 1350000 | slow |
| 2 | 1350001 | 1375000 | slow |
| 2 | 1375001 | 1400000 | slow |
| 2 | 1400001 | 1425000 | slow |
| 2 | 1425001 | 1450000 | slow |
| 2 | 1450001 | 1475000 | slow |

| | | | |
|---|---|---|---|
| 2 | 1475001 | 1500000 | slow |
| 2 | 1500001 | 1525000 | slow |
| 2 | 1525001 | 1550000 | slow |
| 2 | 1550001 | 1575000 | slow |
| 2 | 1575001 | 1600000 | slow |
| 2 | 1600001 | 1625000 | slow |
| 2 | 1625001 | 1650000 | slow |
| 2 | 1650001 | 1675000 | slow |
| 2 | 1675001 | 1700000 | slow |
| 2 | 1700001 | 1725000 | slow |
| 2 | 1725001 | 1750000 | slow |
| 2 | 1750001 | 1775000 | slow |
| 2 | 1775001 | 1800000 | slow |
| 2 | 1800001 | 1825000 | slow |
| 2 | 1825001 | 1850000 | slow |
| 2 | 1850001 | 1875000 | slow |
| 2 | 1875001 | 1900000 | slow |
| 2 | 1900001 | 1925000 | slow |
| 2 | 1925001 | 1950000 | slow |
| 2 | 1950001 | 1975000 | slow |
| 2 | 1975001 | 2000000 | slow |
| 2 | 2000001 | 2025000 | slow |
| 2 | 2025001 | 2050000 | slow |
| 2 | 2050001 | 2075000 | slow |
| 2 | 2075001 | 2100000 | slow |
| 2 | 2100001 | 2125000 | slow |
| 2 | 2125001 | 2150000 | slow |
| 2 | 2150001 | 2175000 | slow |
| 2 | 2175001 | 2200000 | slow |
| 2 | 2200001 | 2225000 | slow |
| 2 | 2225001 | 2250000 | slow |
| 2 | 2250001 | 2275000 | slow |
| 2 | 2275001 | 2300000 | slow |
| 2 | 2300001 | 2325000 | slow |
| 2 | 2325001 | 2350000 | slow |
| 2 | 2350001 | 2375000 | slow |
| 2 | 2375001 | 2400000 | slow |
| 2 | 2400001 | 2425000 | slow |
| 2 | 2425001 | 2450000 | slow |
| 2 | 2450001 | 2475000 | slow |
| 2 | 2475001 | 2500000 | slow |
| 2 | 2500001 | 2525000 | slow |
| 2 | 2525001 | 2550000 | slow |
| 2 | 2550001 | 2575000 | slow |
| 2 | 2575001 | 2600000 | slow |
| 2 | 2600001 | 2625000 | slow |
| 2 | 2625001 | 2650000 | slow |
| 2 | 2650001 | 2675000 | slow |

| | | | |
|---|---|---|---|
| 2 | 2675001 | 2700000 | slow |
| 2 | 2700001 | 2725000 | slow |
| 2 | 2725001 | 2750000 | slow |
| 2 | 2750001 | 2775000 | slow |
| 2 | 2775001 | 2800000 | fast |
| 2 | 2800001 | 2825000 | fast |
| 2 | 2825001 | 2850000 | fast |
| 2 | 2850001 | 2875000 | fast |
| 2 | 2875001 | 2900000 | fast |
| 2 | 2900001 | 2925000 | fast |
| 2 | 2925001 | 2950000 | fast |
| 2 | 2950001 | 2975000 | slow |
| 2 | 2975001 | 3000000 | slow |
| 2 | 3000001 | 3025000 | slow |
| 2 | 3025001 | 3050000 | slow |
| 2 | 3050001 | 3075000 | slow |
| 2 | 3075001 | 3100000 | slow |
| 2 | 3100001 | 3125000 | slow |
| 2 | 3125001 | 3150000 | slow |
| 2 | 3150001 | 3175000 | slow |
| 2 | 3175001 | 3200000 | slow |
| 2 | 3200001 | 3225000 | slow |
| 2 | 3225001 | 3250000 | slow |
| 2 | 3250001 | 3275000 | fast |
| 2 | 3275001 | 3300000 | fast |
| 2 | 3300001 | 3325000 | fast |
| 2 | 3325001 | 3350000 | fast |
| 2 | 3350001 | 3375000 | slow |
| 2 | 3375001 | 3400000 | slow |
| 2 | 3400001 | 3425000 | slow |
| 2 | 3425001 | 3450000 | slow |
| 2 | 3450001 | 3475000 | fast |
| 2 | 3475001 | 3500000 | fast |
| 2 | 3500001 | 3525000 | fast |
| 2 | 3525001 | 3550000 | fast |
| 2 | 3550001 | 3575000 | fast |
| 2 | 3575001 | 3600000 | fast |
| 2 | 3600001 | 3625000 | fast |
| 2 | 3625001 | 3650000 | fast |
| 2 | 3650001 | 3675000 | fast |
| 2 | 3675001 | 3700000 | fast |
| 2 | 3700001 | 3725000 | fast |
| 2 | 3725001 | 3750000 | fast |
| 2 | 3750001 | 3775000 | fast |
| 2 | 3775001 | 3800000 | fast |
| 2 | 3800001 | 3825000 | fast |
| 2 | 3825001 | 3850000 | fast |

| | | | |
|---|---|---|---|
| 2 | 3850001 | 3875000 | fast |
| 2 | 3875001 | 3900000 | fast |
| 2 | 3900001 | 3925000 | fast |
| 2 | 3925001 | 3950000 | fast |
| 2 | 3950001 | 3975000 | fast |
| 2 | 3975001 | 4000000 | fast |
| 2 | 4000001 | 4025000 | fast |
| 2 | 4025001 | 4050000 | fast |
| 2 | 4050001 | 4075000 | fast |
| 2 | 4075001 | 4100000 | fast |
| 2 | 4100001 | 4125000 | fast |
| 2 | 4125001 | 4150000 | fast |
| 2 | 4150001 | 4175000 | fast |
| 2 | 4175001 | 4200000 | fast |
| 2 | 4200001 | 4225000 | fast |
| 2 | 4225001 | 4250000 | fast |
| 2 | 4250001 | 4275000 | fast |
| 2 | 4275001 | 4300000 | fast |
| 2 | 4300001 | 4325000 | fast |
| 2 | 4325001 | 4350000 | fast |
| 2 | 4350001 | 4375000 | fast |
| 2 | 4375001 | 4400000 | fast |
| 2 | 4400001 | 4425000 | fast |
| 2 | 4425001 | 4450000 | fast |
| 2 | 4450001 | 4475000 | fast |
| 2 | 4475001 | 4500000 | fast |
| 2 | 4500001 | 4525000 | fast |
| 2 | 4525001 | 4550000 | fast |
| 2 | 4550001 | 4575000 | fast |
| 2 | 4575001 | 4600000 | fast |
| 2 | 4600001 | 4625000 | fast |
| 2 | 4625001 | 4650000 | fast |
| 2 | 4650001 | 4675000 | fast |
| 2 | 4675001 | 4700000 | fast |
| 2 | 4700001 | 4725000 | fast |
| 2 | 4725001 | 4750000 | fast |
| 2 | 4750001 | 4775000 | fast |
| 2 | 4775001 | 4800000 | fast |
| 2 | 4800001 | 4825000 | fast |
| 2 | 4825001 | 4850000 | fast |
| 2 | 4850001 | 4875000 | fast |
| 2 | 4875001 | 4900000 | fast |
| 2 | 4900001 | 4925000 | fast |
| 2 | 4925001 | 4950000 | fast |
| 2 | 4950001 | 4975000 | fast |
| 2 | 4975001 | 5000000 | fast |
| 2 | 5000001 | 5025000 | fast |
| 2 | 5025001 | 5050000 | fast |

| | | | |
|---|---|---|---|
| 2 | 5050001 | 5075000 | fast |
| 2 | 5075001 | 5100000 | fast |
| 2 | 5100001 | 5125000 | fast |
| 2 | 5125001 | 5150000 | fast |
| 2 | 5150001 | 5175000 | fast |
| 2 | 5175001 | 5200000 | fast |
| 2 | 5200001 | 5225000 | fast |
| 2 | 5225001 | 5250000 | fast |
| 2 | 5250001 | 5275000 | fast |
| 2 | 5275001 | 5300000 | fast |
| 2 | 5300001 | 5325000 | fast |
| 2 | 5325001 | 5350000 | fast |
| 2 | 5350001 | 5375000 | fast |
| 2 | 5375001 | 5400000 | fast |
| 2 | 5400001 | 5425000 | fast |
| 2 | 5425001 | 5450000 | fast |
| 2 | 5450001 | 5475000 | fast |
| 2 | 5475001 | 5500000 | fast |
| 2 | 5500001 | 5525000 | fast |
| 2 | 5525001 | 5550000 | fast |
| 2 | 5550001 | 5575000 | fast |
| 2 | 5575001 | 5600000 | fast |
| 2 | 5600001 | 5625000 | fast |
| 2 | 5625001 | 5650000 | fast |
| 2 | 5650001 | 5675000 | fast |
| 2 | 5675001 | 5700000 | fast |
| 2 | 5700001 | 5725000 | fast |
| 2 | 5725001 | 5750000 | fast |
| 2 | 5750001 | 5775000 | fast |
| 2 | 5775001 | 5800000 | fast |
| 2 | 5800001 | 5825000 | fast |
| 2 | 5825001 | 5850000 | fast |
| 2 | 5850001 | 5875000 | fast |
| 2 | 5875001 | 5900000 | fast |
| 2 | 5900001 | 5925000 | fast |
| 2 | 5925001 | 5950000 | fast |
| 2 | 5950001 | 5975000 | fast |
| 2 | 5975001 | 6000000 | fast |
| 2 | 6000001 | 6025000 | fast |
| 2 | 6025001 | 6050000 | fast |
| 2 | 6050001 | 6075000 | fast |
| 2 | 6075001 | 6100000 | fast |
| 2 | 6100001 | 6125000 | fast |
| 2 | 6125001 | 6150000 | fast |
| 2 | 6150001 | 6175000 | fast |
| 2 | 6175001 | 6200000 | fast |
| 2 | 6200001 | 6225000 | fast |

| | | | |
|---|---|---|---|
| 2 | 6225001 | 6250000 | fast |
| 2 | 6250001 | 6275000 | fast |
| 2 | 6275001 | 6300000 | fast |
| 2 | 6300001 | 6325000 | fast |
| 2 | 6325001 | 6350000 | fast |
| 2 | 6350001 | 6375000 | fast |
| 2 | 6375001 | 6400000 | fast |
| 2 | 6400001 | 6425000 | fast |
| 2 | 6425001 | 6450000 | fast |
| 2 | 6450001 | 6475000 | fast |
| 2 | 6475001 | 6500000 | fast |
| 2 | 6500001 | 6525000 | fast |
| 2 | 6525001 | 6550000 | fast |
| 2 | 6550001 | 6575000 | fast |
| 2 | 6575001 | 6600000 | fast |
| 2 | 6600001 | 6625000 | fast |
| 2 | 6625001 | 6650000 | fast |
| 2 | 6650001 | 6675000 | fast |
| 2 | 6675001 | 6700000 | fast |
| 2 | 6700001 | 6725000 | slow |
| 2 | 6725001 | 6750000 | slow |
| 2 | 6750001 | 6775000 | slow |
| 2 | 6775001 | 6800000 | slow |
| 2 | 6800001 | 6825000 | fast |
| 2 | 6825001 | 6850000 | fast |
| 2 | 6850001 | 6875000 | fast |
| 2 | 6875001 | 6900000 | slow |
| 2 | 6900001 | 6925000 | slow |
| 2 | 6925001 | 6950000 | slow |
| 2 | 6950001 | 6975000 | slow |
| 2 | 6975001 | 7000000 | slow |
| 2 | 7000001 | 7025000 | slow |
| 2 | 7025001 | 7050000 | slow |
| 2 | 7050001 | 7075000 | slow |
| 2 | 7075001 | 7100000 | slow |
| 2 | 7100001 | 7125000 | slow |
| 2 | 7125001 | 7150000 | slow |
| 2 | 7150001 | 7175000 | slow |
| 2 | 7175001 | 7200000 | slow |
| 2 | 7200001 | 7225000 | slow |
| 2 | 7225001 | 7250000 | slow |
| 2 | 7250001 | 7275000 | slow |
| 2 | 7275001 | 7300000 | slow |
| 2 | 7300001 | 7325000 | slow |
| 2 | 7325001 | 7350000 | slow |
| 2 | 7350001 | 7375000 | slow |
| 2 | 7375001 | 7400000 | slow |
| 2 | 7400001 | 7425000 | slow |

| | | | |
|---|---|---|---|
| 2 | 7425001 | 7450000 | slow |
| 2 | 7450001 | 7475000 | slow |
| 2 | 7475001 | 7500000 | slow |
| 2 | 7500001 | 7525000 | slow |
| 2 | 7525001 | 7550000 | slow |
| 2 | 7550001 | 7575000 | slow |
| 2 | 7575001 | 7600000 | slow |
| 2 | 7600001 | 7625000 | fast |
| 2 | 7625001 | 7650000 | slow |
| 2 | 7650001 | 7675000 | slow |
| 2 | 7675001 | 7700000 | slow |
| 2 | 7700001 | 7725000 | slow |
| 2 | 7725001 | 7750000 | slow |
| 2 | 7750001 | 7775000 | slow |
| 2 | 7775001 | 7800000 | slow |
| 2 | 7800001 | 7825000 | fast |
| 2 | 7825001 | 7850000 | fast |
| 2 | 7850001 | 7875000 | fast |
| 2 | 7875001 | 7900000 | slow |
| 2 | 7900001 | 7925000 | slow |
| 2 | 7925001 | 7950000 | slow |
| 2 | 7950001 | 7975000 | slow |
| 2 | 7975001 | 8000000 | slow |
| 2 | 8000001 | 8025000 | fast |
| 2 | 8025001 | 8050000 | fast |
| 2 | 8050001 | 8075000 | fast |
| 2 | 8075001 | 8100000 | fast |
| 2 | 8100001 | 8125000 | fast |
| 2 | 8125001 | 8150000 | fast |
| 2 | 8150001 | 8175000 | fast |
| 2 | 8175001 | 8200000 | fast |
| 2 | 8200001 | 8225000 | fast |
| 2 | 8225001 | 8250000 | fast |
| 2 | 8250001 | 8275000 | fast |
| 2 | 8275001 | 8300000 | fast |
| 2 | 8300001 | 8325000 | fast |
| 2 | 8325001 | 8350000 | fast |
| 2 | 8350001 | 8375000 | fast |
| 2 | 8375001 | 8400000 | fast |
| 2 | 8400001 | 8425000 | fast |
| 2 | 8425001 | 8450000 | fast |
| 2 | 8450001 | 8475000 | fast |
| 2 | 8475001 | 8500000 | fast |
| 2 | 8500001 | 8525000 | fast |
| 2 | 8525001 | 8550000 | fast |
| 2 | 8550001 | 8575000 | fast |
| 2 | 8575001 | 8600000 | fast |

| | | | |
|---|---|---|---|
| 2 | 8600001 | 8625000 | fast |
| 2 | 8625001 | 8650000 | fast |
| 2 | 8650001 | 8675000 | fast |
| 2 | 8675001 | 8700000 | fast |
| 2 | 8700001 | 8725000 | fast |
| 2 | 8725001 | 8750000 | fast |
| 2 | 8750001 | 8775000 | fast |
| 2 | 8775001 | 8800000 | fast |
| 2 | 8800001 | 8825000 | fast |
| 2 | 8825001 | 8850000 | fast |
| 2 | 8850001 | 8875000 | fast |
| 2 | 8875001 | 8900000 | fast |
| 2 | 8900001 | 8925000 | fast |
| 2 | 8925001 | 8950000 | fast |
| 2 | 8950001 | 8975000 | fast |
| 2 | 8975001 | 9000000 | fast |
| 3 | 1 | 25000 | fast |
| 3 | 25001 | 50000 | fast |
| 3 | 50001 | 75000 | fast |
| 3 | 75001 | 100000 | fast |
| 3 | 100001 | 125000 | fast |
| 3 | 125001 | 150000 | fast |
| 3 | 150001 | 175000 | fast |
| 3 | 175001 | 200000 | fast |
| 3 | 200001 | 225000 | fast |
| 3 | 225001 | 250000 | fast |
| 3 | 250001 | 275000 | fast |
| 3 | 275001 | 300000 | fast |
| 3 | 300001 | 325000 | fast |
| 3 | 325001 | 350000 | fast |
| 3 | 350001 | 375000 | fast |
| 3 | 375001 | 400000 | fast |
| 3 | 400001 | 425000 | fast |
| 3 | 425001 | 450000 | fast |
| 3 | 450001 | 475000 | fast |
| 3 | 475001 | 500000 | fast |
| 3 | 500001 | 525000 | fast |
| 3 | 525001 | 550000 | fast |
| 3 | 550001 | 575000 | slow |
| 3 | 575001 | 600000 | slow |
| 3 | 600001 | 625000 | slow |
| 3 | 625001 | 650000 | slow |
| 3 | 650001 | 675000 | fast |
| 3 | 675001 | 700000 | slow |
| 3 | 700001 | 725000 | slow |
| 3 | 725001 | 750000 | slow |
| 3 | 750001 | 775000 | slow |
| 3 | 775001 | 800000 | slow |

| | | | |
|---|---|---|---|
| 3 | 800001 | 825000 | slow |
| 3 | 825001 | 850000 | slow |
| 3 | 850001 | 875000 | slow |
| 3 | 875001 | 900000 | slow |
| 3 | 900001 | 925000 | slow |
| 3 | 925001 | 950000 | slow |
| 3 | 950001 | 975000 | slow |
| 3 | 975001 | 1000000 | slow |
| 3 | 1000001 | 1025000 | slow |
| 3 | 1025001 | 1050000 | slow |
| 3 | 1050001 | 1075000 | slow |
| 3 | 1075001 | 1100000 | slow |
| 3 | 1100001 | 1125000 | slow |
| 3 | 1125001 | 1150000 | slow |
| 3 | 1150001 | 1175000 | slow |
| 3 | 1175001 | 1200000 | slow |
| 3 | 1200001 | 1225000 | slow |
| 3 | 1225001 | 1250000 | slow |
| 3 | 1250001 | 1275000 | slow |
| 3 | 1275001 | 1300000 | slow |
| 3 | 1300001 | 1325000 | slow |
| 3 | 1325001 | 1350000 | slow |
| 3 | 1350001 | 1375000 | slow |
| 3 | 1375001 | 1400000 | slow |
| 3 | 1400001 | 1425000 | slow |
| 3 | 1425001 | 1450000 | slow |
| 3 | 1450001 | 1475000 | slow |
| 3 | 1475001 | 1500000 | slow |
| 3 | 1500001 | 1525000 | slow |
| 3 | 1525001 | 1550000 | slow |
| 3 | 1550001 | 1575000 | slow |
| 3 | 1575001 | 1600000 | slow |
| 3 | 1600001 | 1625000 | slow |
| 3 | 1625001 | 1650000 | slow |
| 3 | 1650001 | 1675000 | slow |
| 3 | 1675001 | 1700000 | slow |
| 3 | 1700001 | 1725000 | slow |
| 3 | 1725001 | 1750000 | slow |
| 3 | 1750001 | 1775000 | slow |
| 3 | 1775001 | 1800000 | slow |
| 3 | 1800001 | 1825000 | slow |
| 3 | 1825001 | 1850000 | slow |
| 3 | 1850001 | 1875000 | slow |
| 3 | 1875001 | 1900000 | slow |
| 3 | 1900001 | 1925000 | slow |
| 3 | 1925001 | 1950000 | slow |
| 3 | 1950001 | 1975000 | slow |

| | | | |
|---|---|---|---|
| 3 | 1975001 | 2000000 | slow |
| 3 | 2000001 | 2025000 | slow |
| 3 | 2025001 | 2050000 | slow |
| 3 | 2050001 | 2075000 | slow |
| 3 | 2075001 | 2100000 | slow |
| 3 | 2100001 | 2125000 | slow |
| 3 | 2125001 | 2150000 | slow |
| 3 | 2150001 | 2175000 | slow |
| 3 | 2175001 | 2200000 | slow |
| 3 | 2200001 | 2225000 | slow |
| 3 | 2225001 | 2250000 | slow |
| 3 | 2250001 | 2275000 | slow |
| 3 | 2275001 | 2300000 | slow |
| 3 | 2300001 | 2325000 | slow |
| 3 | 2325001 | 2350000 | slow |
| 3 | 2350001 | 2375000 | slow |
| 3 | 2375001 | 2400000 | slow |
| 3 | 2400001 | 2425000 | slow |
| 3 | 2425001 | 2450000 | slow |
| 3 | 2450001 | 2475000 | slow |
| 3 | 2475001 | 2500000 | slow |
| 3 | 2500001 | 2525000 | slow |
| 3 | 2525001 | 2550000 | slow |
| 3 | 2550001 | 2575000 | slow |
| 3 | 2575001 | 2600000 | slow |
| 3 | 2600001 | 2625000 | slow |
| 3 | 2625001 | 2650000 | slow |
| 3 | 2650001 | 2675000 | slow |
| 3 | 2675001 | 2700000 | slow |
| 3 | 2700001 | 2725000 | slow |
| 3 | 2725001 | 2750000 | slow |
| 3 | 2750001 | 2775000 | slow |
| 3 | 2775001 | 2800000 | slow |
| 3 | 2800001 | 2825000 | slow |
| 3 | 2825001 | 2850000 | slow |
| 3 | 2850001 | 2875000 | slow |
| 3 | 2875001 | 2900000 | slow |
| 3 | 2900001 | 2925000 | slow |
| 3 | 2925001 | 2950000 | slow |
| 3 | 2950001 | 2975000 | slow |
| 3 | 2975001 | 3000000 | slow |
| 3 | 3000001 | 3025000 | slow |
| 3 | 3025001 | 3050000 | slow |
| 3 | 3050001 | 3075000 | slow |
| 3 | 3075001 | 3100000 | slow |
| 3 | 3100001 | 3125000 | slow |
| 3 | 3125001 | 3150000 | slow |
| 3 | 3150001 | 3175000 | fast |

| | | | |
|---|---|---|---|
| 3 | 3175001 | 3200000 | slow |
| 3 | 3200001 | 3225000 | slow |
| 3 | 3225001 | 3250000 | slow |
| 3 | 3250001 | 3275000 | slow |
| 3 | 3275001 | 3300000 | fast |
| 3 | 3300001 | 3325000 | fast |
| 3 | 3325001 | 3350000 | slow |
| 3 | 3350001 | 3375000 | slow |
| 3 | 3375001 | 3400000 | fast |
| 3 | 3400001 | 3425000 | fast |
| 3 | 3425001 | 3450000 | fast |
| 3 | 3450001 | 3475000 | fast |
| 3 | 3475001 | 3500000 | fast |
| 3 | 3500001 | 3525000 | fast |
| 3 | 3525001 | 3550000 | fast |
| 3 | 3550001 | 3575000 | fast |
| 3 | 3575001 | 3600000 | fast |
| 3 | 3600001 | 3625000 | fast |
| 3 | 3625001 | 3650000 | fast |
| 3 | 3650001 | 3675000 | slow |
| 3 | 3675001 | 3700000 | slow |
| 3 | 3700001 | 3725000 | slow |
| 3 | 3725001 | 3750000 | slow |
| 3 | 3750001 | 3775000 | slow |
| 3 | 3775001 | 3800000 | slow |
| 3 | 3800001 | 3825000 | slow |
| 3 | 3825001 | 3850000 | slow |
| 3 | 3850001 | 3875000 | slow |
| 3 | 3875001 | 3900000 | slow |
| 3 | 3900001 | 3925000 | slow |
| 3 | 3925001 | 3950000 | slow |
| 3 | 3950001 | 3975000 | slow |
| 3 | 3975001 | 4000000 | slow |
| 3 | 4000001 | 4025000 | slow |
| 3 | 4025001 | 4050000 | slow |
| 3 | 4050001 | 4075000 | slow |
| 3 | 4075001 | 4100000 | slow |
| 3 | 4100001 | 4125000 | slow |
| 3 | 4125001 | 4150000 | slow |
| 3 | 4150001 | 4175000 | slow |
| 3 | 4175001 | 4200000 | slow |
| 3 | 4200001 | 4225000 | slow |
| 3 | 4225001 | 4250000 | slow |
| 3 | 4250001 | 4275000 | slow |
| 3 | 4275001 | 4300000 | slow |
| 3 | 4300001 | 4325000 | slow |
| 3 | 4325001 | 4350000 | slow |

| | | | |
|---|---|---|---|
| 3 | 4350001 | 4375000 | slow |
| 3 | 4375001 | 4400000 | slow |
| 3 | 4400001 | 4425000 | slow |
| 3 | 4425001 | 4450000 | slow |
| 3 | 4450001 | 4475000 | slow |
| 3 | 4475001 | 4500000 | slow |
| 3 | 4500001 | 4525000 | slow |
| 3 | 4525001 | 4550000 | slow |
| 3 | 4550001 | 4575000 | slow |
| 3 | 4575001 | 4600000 | slow |
| 3 | 4600001 | 4625000 | slow |
| 3 | 4625001 | 4650000 | slow |
| 3 | 4650001 | 4675000 | slow |
| 3 | 4675001 | 4700000 | slow |
| 3 | 4700001 | 4725000 | slow |
| 3 | 4725001 | 4750000 | slow |
| 3 | 4750001 | 4775000 | slow |
| 3 | 4775001 | 4800000 | slow |
| 3 | 4800001 | 4825000 | slow |
| 3 | 4825001 | 4850000 | slow |
| 3 | 4850001 | 4875000 | slow |
| 3 | 4875001 | 4900000 | slow |
| 3 | 4900001 | 4925000 | slow |
| 3 | 4925001 | 4950000 | slow |
| 3 | 4950001 | 4975000 | slow |
| 3 | 4975001 | 5000000 | slow |
| 3 | 5000001 | 5025000 | slow |
| 3 | 5025001 | 5050000 | slow |
| 3 | 5050001 | 5075000 | slow |
| 3 | 5075001 | 5100000 | slow |
| 3 | 5100001 | 5125000 | slow |
| 3 | 5125001 | 5150000 | slow |
| 3 | 5150001 | 5175000 | slow |
| 3 | 5175001 | 5200000 | slow |
| 3 | 5200001 | 5225000 | slow |
| 3 | 5225001 | 5250000 | slow |
| 3 | 5250001 | 5275000 | slow |
| 3 | 5275001 | 5300000 | slow |
| 3 | 5300001 | 5325000 | slow |
| 3 | 5325001 | 5350000 | slow |
| 3 | 5350001 | 5375000 | fast |
| 3 | 5375001 | 5400000 | fast |
| 3 | 5400001 | 5425000 | slow |
| 3 | 5425001 | 5450000 | slow |
| 3 | 5450001 | 5475000 | slow |
| 3 | 5475001 | 5500000 | slow |
| 3 | 5500001 | 5525000 | slow |
| 3 | 5525001 | 5550000 | slow |

| | | | |
|---|---|---|---|
| 3 | 5550001 | 5575000 | slow |
| 3 | 5575001 | 5600000 | slow |
| 3 | 5600001 | 5625000 | slow |
| 3 | 5625001 | 5650000 | slow |
| 3 | 5650001 | 5675000 | slow |
| 3 | 5675001 | 5700000 | slow |
| 3 | 5700001 | 5725000 | slow |
| 3 | 5725001 | 5750000 | slow |
| 3 | 5750001 | 5775000 | slow |
| 3 | 5775001 | 5800000 | slow |
| 3 | 5800001 | 5825000 | slow |
| 3 | 5825001 | 5850000 | slow |
| 3 | 5850001 | 5875000 | slow |
| 3 | 5875001 | 5900000 | slow |
| 3 | 5900001 | 5925000 | slow |
| 3 | 5925001 | 5950000 | slow |
| 3 | 5950001 | 5975000 | slow |
| 3 | 5975001 | 6000000 | slow |
| 3 | 6000001 | 6025000 | slow |
| 3 | 6025001 | 6050000 | slow |
| 3 | 6050001 | 6075000 | slow |
| 3 | 6075001 | 6100000 | slow |
| 3 | 6100001 | 6125000 | slow |
| 3 | 6125001 | 6150000 | slow |
| 3 | 6150001 | 6175000 | slow |
| 3 | 6175001 | 6200000 | slow |
| 3 | 6200001 | 6225000 | slow |
| 3 | 6225001 | 6250000 | slow |
| 3 | 6250001 | 6275000 | slow |
| 3 | 6275001 | 6300000 | fast |
| 3 | 6300001 | 6325000 | fast |
| 3 | 6325001 | 6350000 | fast |
| 3 | 6350001 | 6375000 | fast |
| 3 | 6375001 | 6400000 | fast |
| 3 | 6400001 | 6425000 | fast |
| 3 | 6425001 | 6450000 | fast |
| 3 | 6450001 | 6475000 | fast |
| 3 | 6475001 | 6500000 | fast |
| 3 | 6500001 | 6525000 | fast |
| 3 | 6525001 | 6550000 | fast |
| 3 | 6550001 | 6575000 | fast |
| 3 | 6575001 | 6600000 | fast |
| 3 | 6600001 | 6625000 | fast |
| 3 | 6625001 | 6650000 | fast |
| 3 | 6650001 | 6675000 | fast |
| 3 | 6675001 | 6700000 | fast |
| 3 | 6700001 | 6725000 | fast |

| | | | |
|---|---|---|---|
| 3 | 6725001 | 6750000 | fast |
| 3 | 6750001 | 6775000 | fast |
| 3 | 6775001 | 6800000 | fast |
| 3 | 6800001 | 6825000 | fast |
| 3 | 6825001 | 6850000 | fast |
| 3 | 6850001 | 6875000 | fast |
| 3 | 6875001 | 6900000 | fast |
| 3 | 6900001 | 6925000 | fast |
| 3 | 6925001 | 6950000 | fast |
| 3 | 6950001 | 6975000 | fast |
| 3 | 6975001 | 7000000 | fast |
| 3 | 7000001 | 7025000 | fast |
| 3 | 7025001 | 7050000 | fast |
| 3 | 7050001 | 7075000 | fast |
| 3 | 7075001 | 7100000 | fast |
| 3 | 7100001 | 7125000 | fast |
| 3 | 7125001 | 7150000 | fast |
| 3 | 7150001 | 7175000 | fast |
| 3 | 7175001 | 7200000 | fast |
| 3 | 7200001 | 7225000 | fast |
| 3 | 7225001 | 7250000 | fast |
| 3 | 7250001 | 7275000 | fast |
| 3 | 7275001 | 7300000 | fast |
| 3 | 7300001 | 7325000 | fast |
| 3 | 7325001 | 7350000 | fast |
| 3 | 7350001 | 7375000 | fast |
| 3 | 7375001 | 7400000 | fast |
| 3 | 7400001 | 7425000 | fast |
| 3 | 7425001 | 7450000 | fast |
| 3 | 7450001 | 7475000 | fast |
| 3 | 7475001 | 7500000 | fast |
| 3 | 7500001 | 7525000 | fast |
| 3 | 7525001 | 7550000 | fast |
| 3 | 7550001 | 7575000 | fast |
| 3 | 7575001 | 7600000 | fast |
| 3 | 7600001 | 7625000 | fast |
| 3 | 7625001 | 7650000 | fast |
| 3 | 7650001 | 7675000 | fast |
| 3 | 7675001 | 7700000 | fast |
| 3 | 7700001 | 7725000 | fast |
| 3 | 7725001 | 7750000 | fast |
| 3 | 7750001 | 7775000 | fast |
| 3 | 7775001 | 7800000 | fast |
| 4 | 1 | 25000 | fast |
| 4 | 25001 | 50000 | fast |
| 4 | 50001 | 75000 | fast |
| 4 | 75001 | 100000 | fast |
| 4 | 100001 | 125000 | fast |

| | | | |
|---|---|---|---|
| 4 | 125001 | 150000 | fast |
| 4 | 150001 | 175000 | fast |
| 4 | 175001 | 200000 | fast |
| 4 | 200001 | 225000 | fast |
| 4 | 225001 | 250000 | fast |
| 4 | 250001 | 275000 | fast |
| 4 | 275001 | 300000 | fast |
| 4 | 300001 | 325000 | fast |
| 4 | 325001 | 350000 | fast |
| 4 | 350001 | 375000 | fast |
| 4 | 375001 | 400000 | fast |
| 4 | 400001 | 425000 | fast |
| 4 | 425001 | 450000 | fast |
| 4 | 450001 | 475000 | fast |
| 4 | 475001 | 500000 | fast |
| 4 | 500001 | 525000 | fast |
| 4 | 525001 | 550000 | slow |
| 4 | 550001 | 575000 | slow |
| 4 | 575001 | 600000 | slow |
| 4 | 600001 | 625000 | slow |
| 4 | 625001 | 650000 | slow |
| 4 | 650001 | 675000 | slow |
| 4 | 675001 | 700000 | slow |
| 4 | 700001 | 725000 | slow |
| 4 | 725001 | 750000 | slow |
| 4 | 750001 | 775000 | slow |
| 4 | 775001 | 800000 | slow |
| 4 | 800001 | 825000 | slow |
| 4 | 825001 | 850000 | slow |
| 4 | 850001 | 875000 | slow |
| 4 | 875001 | 900000 | slow |
| 4 | 900001 | 925000 | slow |
| 4 | 925001 | 950000 | slow |
| 4 | 950001 | 975000 | slow |
| 4 | 975001 | 1000000 | slow |
| 4 | 1000001 | 1025000 | slow |
| 4 | 1025001 | 1050000 | slow |
| 4 | 1050001 | 1075000 | slow |
| 4 | 1075001 | 1100000 | slow |
| 4 | 1100001 | 1125000 | slow |
| 4 | 1125001 | 1150000 | slow |
| 4 | 1150001 | 1175000 | slow |
| 4 | 1175001 | 1200000 | slow |
| 4 | 1200001 | 1225000 | slow |
| 4 | 1225001 | 1250000 | slow |
| 4 | 1250001 | 1275000 | slow |
| 4 | 1275001 | 1300000 | slow |

| | | | |
|---|---|---|---|
| 4 | 1300001 | 1325000 | slow |
| 4 | 1325001 | 1350000 | slow |
| 4 | 1350001 | 1375000 | slow |
| 4 | 1375001 | 1400000 | slow |
| 4 | 1400001 | 1425000 | slow |
| 4 | 1425001 | 1450000 | slow |
| 4 | 1450001 | 1475000 | slow |
| 4 | 1475001 | 1500000 | slow |
| 4 | 1500001 | 1525000 | slow |
| 4 | 1525001 | 1550000 | slow |
| 4 | 1550001 | 1575000 | slow |
| 4 | 1575001 | 1600000 | slow |
| 4 | 1600001 | 1625000 | fast |
| 4 | 1625001 | 1650000 | slow |
| 4 | 1650001 | 1675000 | slow |
| 4 | 1675001 | 1700000 | slow |
| 4 | 1700001 | 1725000 | slow |
| 4 | 1725001 | 1750000 | slow |
| 4 | 1750001 | 1775000 | slow |
| 4 | 1775001 | 1800000 | slow |
| 4 | 1800001 | 1825000 | slow |
| 4 | 1825001 | 1850000 | slow |
| 4 | 1850001 | 1875000 | slow |
| 4 | 1875001 | 1900000 | slow |
| 4 | 1900001 | 1925000 | slow |
| 4 | 1925001 | 1950000 | slow |
| 4 | 1950001 | 1975000 | slow |
| 4 | 1975001 | 2000000 | slow |
| 4 | 2000001 | 2025000 | slow |
| 4 | 2025001 | 2050000 | slow |
| 4 | 2050001 | 2075000 | slow |
| 4 | 2075001 | 2100000 | slow |
| 4 | 2100001 | 2125000 | slow |
| 4 | 2125001 | 2150000 | slow |
| 4 | 2150001 | 2175000 | slow |
| 4 | 2175001 | 2200000 | slow |
| 4 | 2200001 | 2225000 | slow |
| 4 | 2225001 | 2250000 | fast |
| 4 | 2250001 | 2275000 | fast |
| 4 | 2275001 | 2300000 | slow |
| 4 | 2300001 | 2325000 | slow |
| 4 | 2325001 | 2350000 | slow |
| 4 | 2350001 | 2375000 | slow |
| 4 | 2375001 | 2400000 | slow |
| 4 | 2400001 | 2425000 | slow |
| 4 | 2425001 | 2450000 | slow |
| 4 | 2450001 | 2475000 | slow |
| 4 | 2475001 | 2500000 | slow |

| | | | |
|---|---|---|---|
| 4 | 2500001 | 2525000 | slow |
| 4 | 2525001 | 2550000 | slow |
| 4 | 2550001 | 2575000 | slow |
| 4 | 2575001 | 2600000 | slow |
| 4 | 2600001 | 2625000 | slow |
| 4 | 2625001 | 2650000 | slow |
| 4 | 2650001 | 2675000 | slow |
| 4 | 2675001 | 2700000 | slow |
| 4 | 2700001 | 2725000 | slow |
| 4 | 2725001 | 2750000 | fast |
| 4 | 2750001 | 2775000 | fast |
| 4 | 2775001 | 2800000 | fast |
| 4 | 2800001 | 2825000 | fast |
| 4 | 2825001 | 2850000 | slow |
| 4 | 2850001 | 2875000 | slow |
| 4 | 2875001 | 2900000 | slow |
| 4 | 2900001 | 2925000 | slow |
| 4 | 2925001 | 2950000 | slow |
| 4 | 2950001 | 2975000 | slow |
| 4 | 2975001 | 3000000 | slow |
| 4 | 3000001 | 3025000 | slow |
| 4 | 3025001 | 3050000 | slow |
| 4 | 3050001 | 3075000 | slow |
| 4 | 3075001 | 3100000 | slow |
| 4 | 3100001 | 3125000 | slow |
| 4 | 3125001 | 3150000 | slow |
| 4 | 3150001 | 3175000 | slow |
| 4 | 3175001 | 3200000 | slow |
| 4 | 3200001 | 3225000 | slow |
| 4 | 3225001 | 3250000 | fast |
| 4 | 3250001 | 3275000 | fast |
| 4 | 3275001 | 3300000 | slow |
| 4 | 3300001 | 3325000 | slow |
| 4 | 3325001 | 3350000 | slow |
| 4 | 3350001 | 3375000 | slow |
| 4 | 3375001 | 3400000 | slow |
| 4 | 3400001 | 3425000 | slow |
| 4 | 3425001 | 3450000 | slow |
| 4 | 3450001 | 3475000 | slow |
| 4 | 3475001 | 3500000 | slow |
| 4 | 3500001 | 3525000 | slow |
| 4 | 3525001 | 3550000 | slow |
| 4 | 3550001 | 3575000 | slow |
| 4 | 3575001 | 3600000 | slow |
| 4 | 3600001 | 3625000 | slow |
| 4 | 3625001 | 3650000 | slow |
| 4 | 3650001 | 3675000 | slow |

| | | | |
|---|---|---|---|
| 4 | 3675001 | 3700000 | slow |
| 4 | 3700001 | 3725000 | slow |
| 4 | 3725001 | 3750000 | slow |
| 4 | 3750001 | 3775000 | slow |
| 4 | 3775001 | 3800000 | fast |
| 4 | 3800001 | 3825000 | fast |
| 4 | 3825001 | 3850000 | fast |
| 4 | 3850001 | 3875000 | fast |
| 4 | 3875001 | 3900000 | fast |
| 4 | 3900001 | 3925000 | fast |
| 4 | 3925001 | 3950000 | fast |
| 4 | 3950001 | 3975000 | fast |
| 4 | 3975001 | 4000000 | fast |
| 4 | 4000001 | 4025000 | fast |
| 4 | 4025001 | 4050000 | fast |
| 4 | 4050001 | 4075000 | fast |
| 4 | 4075001 | 4100000 | fast |
| 4 | 4100001 | 4125000 | fast |
| 4 | 4125001 | 4150000 | fast |
| 4 | 4150001 | 4175000 | fast |
| 4 | 4175001 | 4200000 | fast |
| 4 | 4200001 | 4225000 | fast |
| 4 | 4225001 | 4250000 | fast |
| 4 | 4250001 | 4275000 | fast |
| 4 | 4275001 | 4300000 | fast |
| 4 | 4300001 | 4325000 | fast |
| 4 | 4325001 | 4350000 | fast |
| 4 | 4350001 | 4375000 | fast |
| 4 | 4375001 | 4400000 | fast |
| 4 | 4400001 | 4425000 | fast |
| 4 | 4425001 | 4450000 | fast |
| 4 | 4450001 | 4475000 | fast |
| 4 | 4475001 | 4500000 | fast |
| 4 | 4500001 | 4525000 | fast |
| 4 | 4525001 | 4550000 | fast |
| 4 | 4550001 | 4575000 | fast |
| 4 | 4575001 | 4600000 | fast |
| 4 | 4600001 | 4625000 | fast |
| 4 | 4625001 | 4650000 | fast |
| 4 | 4650001 | 4675000 | slow |
| 4 | 4675001 | 4700000 | slow |
| 4 | 4700001 | 4725000 | slow |
| 4 | 4725001 | 4750000 | slow |
| 4 | 4750001 | 4775000 | slow |
| 4 | 4775001 | 4800000 | slow |
| 4 | 4800001 | 4825000 | slow |
| 4 | 4825001 | 4850000 | slow |
| 4 | 4850001 | 4875000 | slow |

| | | | |
|---|---|---|---|
| 4 | 4875001 | 4900000 | slow |
| 4 | 4900001 | 4925000 | slow |
| 4 | 4925001 | 4950000 | slow |
| 4 | 4950001 | 4975000 | slow |
| 4 | 4975001 | 5000000 | slow |
| 4 | 5000001 | 5025000 | slow |
| 4 | 5025001 | 5050000 | slow |
| 4 | 5050001 | 5075000 | fast |
| 4 | 5075001 | 5100000 | fast |
| 4 | 5100001 | 5125000 | slow |
| 4 | 5125001 | 5150000 | slow |
| 4 | 5150001 | 5175000 | slow |
| 4 | 5175001 | 5200000 | slow |
| 4 | 5200001 | 5225000 | slow |
| 4 | 5225001 | 5250000 | slow |
| 4 | 5250001 | 5275000 | slow |
| 4 | 5275001 | 5300000 | slow |
| 4 | 5300001 | 5325000 | slow |
| 4 | 5325001 | 5350000 | slow |
| 4 | 5350001 | 5375000 | slow |
| 4 | 5375001 | 5400000 | slow |
| 4 | 5400001 | 5425000 | slow |
| 4 | 5425001 | 5450000 | slow |
| 4 | 5450001 | 5475000 | slow |
| 4 | 5475001 | 5500000 | slow |
| 4 | 5500001 | 5525000 | slow |
| 4 | 5525001 | 5550000 | slow |
| 4 | 5550001 | 5575000 | slow |
| 4 | 5575001 | 5600000 | slow |
| 4 | 5600001 | 5625000 | slow |
| 4 | 5625001 | 5650000 | slow |
| 4 | 5650001 | 5675000 | slow |
| 4 | 5675001 | 5700000 | slow |
| 4 | 5700001 | 5725000 | slow |
| 4 | 5725001 | 5750000 | slow |
| 4 | 5750001 | 5775000 | slow |
| 4 | 5775001 | 5800000 | slow |
| 4 | 5800001 | 5825000 | fast |
| 4 | 5825001 | 5850000 | fast |
| 4 | 5850001 | 5875000 | slow |
| 4 | 5875001 | 5900000 | slow |
| 4 | 5900001 | 5925000 | slow |
| 4 | 5925001 | 5950000 | slow |
| 4 | 5950001 | 5975000 | slow |
| 4 | 5975001 | 6000000 | slow |
| 4 | 6000001 | 6025000 | slow |
| 4 | 6025001 | 6050000 | slow |

| | | | |
|---|---|---|---|
| 4 | 6050001 | 6075000 | slow |
| 4 | 6075001 | 6100000 | slow |
| 4 | 6100001 | 6125000 | slow |
| 4 | 6125001 | 6150000 | slow |
| 4 | 6150001 | 6175000 | slow |
| 4 | 6175001 | 6200000 | slow |
| 4 | 6200001 | 6225000 | slow |
| 4 | 6225001 | 6250000 | slow |
| 4 | 6250001 | 6275000 | slow |
| 4 | 6275001 | 6300000 | slow |
| 4 | 6300001 | 6325000 | slow |
| 4 | 6325001 | 6350000 | slow |
| 4 | 6350001 | 6375000 | slow |
| 4 | 6375001 | 6400000 | slow |
| 4 | 6400001 | 6425000 | slow |
| 4 | 6425001 | 6450000 | slow |
| 4 | 6450001 | 6475000 | slow |
| 4 | 6475001 | 6500000 | slow |
| 4 | 6500001 | 6525000 | slow |
| 4 | 6525001 | 6550000 | fast |
| 4 | 6550001 | 6575000 | fast |
| 4 | 6575001 | 6600000 | fast |
| 4 | 6600001 | 6625000 | fast |
| 4 | 6625001 | 6650000 | fast |
| 4 | 6650001 | 6675000 | fast |
| 4 | 6675001 | 6700000 | fast |
| 4 | 6700001 | 6725000 | fast |
| 4 | 6725001 | 6750000 | fast |
| 4 | 6750001 | 6775000 | fast |
| 4 | 6775001 | 6800000 | fast |
| 4 | 6800001 | 6825000 | fast |
| 4 | 6825001 | 6850000 | fast |
| 4 | 6850001 | 6875000 | fast |
| 4 | 6875001 | 6900000 | fast |
| 4 | 6900001 | 6925000 | slow |
| 4 | 6925001 | 6950000 | slow |
| 4 | 6950001 | 6975000 | slow |
| 4 | 6975001 | 7000000 | slow |
| 4 | 7000001 | 7025000 | slow |
| 4 | 7025001 | 7050000 | slow |
| 4 | 7050001 | 7075000 | slow |
| 4 | 7075001 | 7100000 | slow |
| 4 | 7100001 | 7125000 | slow |
| 4 | 7125001 | 7150000 | fast |
| 4 | 7150001 | 7175000 | fast |
| 4 | 7175001 | 7200000 | fast |
| 4 | 7200001 | 7225000 | fast |
| 4 | 7225001 | 7250000 | fast |

| | | | |
|---|---|---|---|
| 4 | 7250001 | 7275000 | fast |
| 4 | 7275001 | 7300000 | fast |
| 4 | 7300001 | 7325000 | fast |
| 4 | 7325001 | 7350000 | fast |
| 4 | 7350001 | 7375000 | fast |
| 4 | 7375001 | 7400000 | fast |
| 4 | 7400001 | 7425000 | fast |
| 4 | 7425001 | 7450000 | fast |
| 4 | 7450001 | 7475000 | fast |
| 4 | 7475001 | 7500000 | fast |
| 4 | 7500001 | 7525000 | fast |
| 4 | 7525001 | 7550000 | fast |
| 4 | 7550001 | 7575000 | fast |
| 4 | 7575001 | 7600000 | fast |
| 4 | 7600001 | 7625000 | fast |
| 4 | 7625001 | 7650000 | fast |
| 4 | 7650001 | 7675000 | fast |
| 4 | 7675001 | 7700000 | fast |
| 4 | 7700001 | 7725000 | fast |
| 4 | 7725001 | 7750000 | fast |
| 4 | 7750001 | 7775000 | fast |
| 4 | 7775001 | 7800000 | fast |
| 4 | 7800001 | 7825000 | fast |
| 4 | 7825001 | 7850000 | fast |
| 4 | 7850001 | 7875000 | fast |
| 4 | 7875001 | 7900000 | fast |
| 4 | 7900001 | 7925000 | fast |
| 4 | 7925001 | 7950000 | fast |
| 4 | 7950001 | 7975000 | fast |
| 4 | 7975001 | 8000000 | fast |
| 4 | 8000001 | 8025000 | fast |
| 4 | 8025001 | 8050000 | slow |
| 4 | 8050001 | 8075000 | slow |
| 4 | 8075001 | 8100000 | slow |
| 4 | 8100001 | 8125000 | slow |
| 4 | 8125001 | 8150000 | slow |
| 4 | 8150001 | 8175000 | slow |
| 4 | 8175001 | 8200000 | slow |
| 4 | 8200001 | 8225000 | slow |
| 4 | 8225001 | 8250000 | slow |
| 4 | 8250001 | 8275000 | slow |
| 4 | 8275001 | 8300000 | slow |
| 4 | 8300001 | 8325000 | slow |
| 4 | 8325001 | 8350000 | slow |
| 4 | 8350001 | 8375000 | slow |
| 4 | 8375001 | 8400000 | slow |
| 4 | 8400001 | 8425000 | slow |

| | | | |
|---|---|---|---|
| 4 | 8425001 | 8450000 | slow |
| 4 | 8450001 | 8475000 | slow |
| 4 | 8475001 | 8500000 | slow |
| 4 | 8500001 | 8525000 | slow |
| 4 | 8525001 | 8550000 | slow |
| 4 | 8550001 | 8575000 | slow |
| 4 | 8575001 | 8600000 | slow |
| 4 | 8600001 | 8625000 | slow |
| 4 | 8625001 | 8650000 | slow |
| 4 | 8650001 | 8675000 | slow |
| 4 | 8675001 | 8700000 | slow |
| 4 | 8700001 | 8725000 | slow |
| 4 | 8725001 | 8750000 | slow |
| 4 | 8750001 | 8775000 | slow |
| 4 | 8775001 | 8800000 | slow |
| 4 | 8800001 | 8825000 | slow |
| 4 | 8825001 | 8850000 | slow |
| 4 | 8850001 | 8875000 | slow |
| 4 | 8875001 | 8900000 | slow |
| 4 | 8900001 | 8925000 | slow |
| 4 | 8925001 | 8950000 | slow |
| 4 | 8950001 | 8975000 | slow |
| 4 | 8975001 | 9000000 | slow |
| 4 | 9000001 | 9025000 | slow |
| 4 | 9025001 | 9050000 | slow |
| 4 | 9050001 | 9075000 | slow |
| 4 | 9075001 | 9100000 | slow |
| 4 | 9100001 | 9125000 | slow |
| 4 | 9125001 | 9150000 | slow |
| 4 | 9150001 | 9175000 | slow |
| 4 | 9175001 | 9200000 | slow |
| 4 | 9200001 | 9225000 | slow |
| 4 | 9225001 | 9250000 | slow |
| 4 | 9250001 | 9275000 | slow |
| 4 | 9275001 | 9300000 | slow |
| 4 | 9300001 | 9325000 | slow |
| 4 | 9325001 | 9350000 | slow |
| 4 | 9350001 | 9375000 | slow |
| 4 | 9375001 | 9400000 | slow |
| 4 | 9400001 | 9425000 | slow |
